# Structure and assembly mechanisms of toxic human islet amyloid polypeptide oligomers associated with copper†
†Electronic supplementary information (ESI) available: Experimental section, Fig. S1–S18 and Tables S1 and S2. See DOI: 10.1039/c6sc00153j


**DOI:** 10.1039/c6sc00153j

**Published:** 2016-05-16

**Authors:** Shin Jung C. Lee, Tae Su Choi, Jong Wha Lee, Hyuck Jin Lee, Dong-Gi Mun, Satoko Akashi, Sang-Won Lee, Mi Hee Lim, Hugh I. Kim

**Affiliations:** a Department of Chemistry , Ulsan National Institute of Science and Technology (UNIST) , Ulsan 44919 , Republic of Korea . Email: mhlim@unist.ac.kr; b Department of Chemistry , Pohang University of Science and Technology (POSTECH) , Pohang 37673 , Republic of Korea; c Department of Chemistry , Research Institute for Natural Sciences , Korea University , Seoul 02841 , Republic of Korea . Email: hughkim@korea.ac.kr; d Graduate School of Medical Life Science , Yokohama City University , Yokohama , Kanagawa 230-0045 , Japan

## Abstract

Amyloidosis is a clinical disorder implicated with the formation of toxic amyloid aggregates. Despite their pathological significance, it is challenging to define the structural characteristics of amyloid oligomers owing to their metastable nature. Herein, we report structural and mechanistic investigations of human islet amyloid polypeptide (hIAPP) oligomers, found in type II diabetes mellitus, in both the absence and presence of disease-relevant metal ions [*i.e.*, Cu(ii) and Zn(ii)]. These metal ions show suppressive effects on hIAPP fibrillation and facilitate the generation of toxic oligomers. Using circular dichroism spectroscopy, transmission electron microscopy, gel electrophoresis, small-angle X-ray scattering, and ion mobility-mass spectrometry, we investigated the assembly mechanisms of hIAPP oligomers in the presence and absence of metal ions. Oligomerization of both metal-free hIAPP and metal-associated hIAPP monomers is initiated following a similar growth model. However, in the presence of Cu(ii), hIAPP monomers self-assemble into small globular aggregates (*R*_g_ ∼ 45 Å) with a random coil structure. This Cu(ii)-associated hIAPP oligomer shows an off-pathway aggregation, and is suggested to be an end product which is toxic to pancreatic β-cells. On the other hand, metal-free hIAPP and Zn(ii)-associated hIAPP monomers generate relatively less toxic aggregates that eventually grow into fibrils. We suggest that the coordination of hIAPP to Cu(ii) and the relatively high stability (*K*_a_, *ca.* 10^8^ M^–1^) of hIAPP–Cu(ii) complexes result in the abnormal conformation and toxicity of hIAPP oligomers. Overall, through combining multiple biophysical methods, our studies suggest that molecular interactions between hIAPP and Cu(ii) induce a different pathway for hIAPP assembly. This work will advance our knowledge of the conformational basis, assembly mechanism, and toxicity of small soluble amyloid oligomers.

## Abstract 2

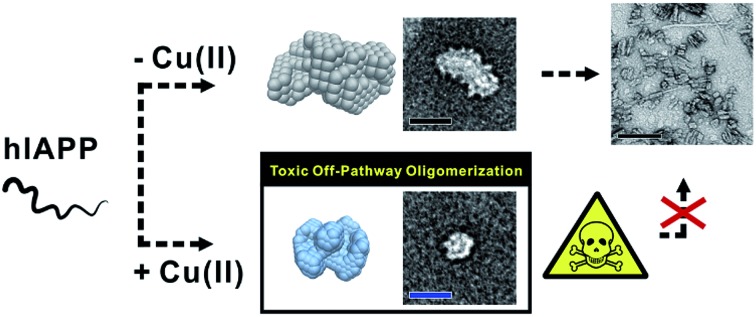
The molecular interaction of hIAPP with Cu(ii) mediates the formation of off-pathway and toxic oligomers which have small-sized and random coil structures.

## Introduction

Amyloid oligomers have generally been considered as the major toxic species in the pathogenesis of amyloidosis.^1^,^2^ In particular, small-sized, soluble oligomers that are formed at the early onset of amyloidosis cause more severe cellular deterioration compared to soluble protein monomers or insoluble fibrils.^2^ Despite the significance of the soluble oligomers in amyloidosis, it is still challenging to characterize the conformation of the oligomers and their correlation to cytotoxicity due to their structural heterogeneity and metastable characteristics.^1^ One of the practical strategies is to adopt specific conditions for stabilizing oligomeric states^3^ or use segments of amyloidogenic proteins which present slow fibrillation kinetics relative to the whole protein.^4^ To date, however, only limited information on soluble oligomers of full-sequence amyloid proteins is available due to the complex behavior of these proteins. Therefore, multiscaled approaches are required to elucidate the structural features and assembly characteristics of the soluble oligomers.

**Fig. 1:**
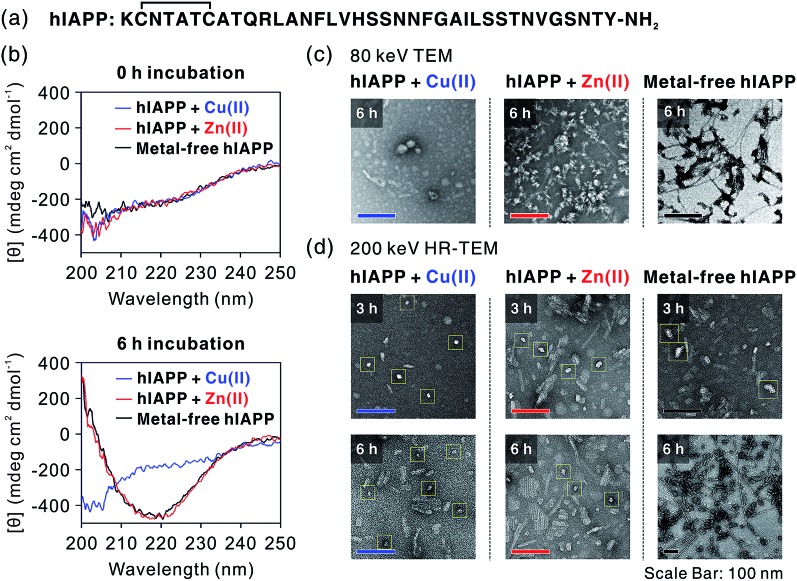
Analysis of hIAPP oligomers by CD and TEM. (a) The sequence of hIAPP. (b) CD spectra of metal-treated and metal-free hIAPP solution after 0 h (top) and 6 h (bottom) incubation. (c) TEM images of hIAPP aggregates. Cu(ii) induces the formation of non-fibrillar aggregates, while Zn(ii)-treated and metal-free hIAPP form amyloid fibrils. (d) HR-TEM images of hIAPP aggregates. Although the oligomerization occurs in all of the 3 h-incubated hIAPP samples, only Cu(ii)-mediated oligomers are stable after 6 h incubation. Zn(ii)-mediated oligomers are mixed with fibrillar aggregates after 6 h incubation. Oligomeric species are marked with yellow boxes.

Aggregates of human islet amyloid polypeptide (hIAPP, Fig. 1a), found in the pancreatic β-cells of type II diabetes mellitus (T2DM) patients, are observed to be linked to T2DM pathogenesis.^5^ To explain the link between hIAPP aggregates and T2DM, several hypotheses regarding the toxicity of hIAPP oligomers have been put forward, including reactive oxygen species (ROS) generation,^6^,^7^ membrane destabilization,^8^,^9^ autophagy dysfunction,^10^ disruption of cell-to-cell interactions,^11^ and secretory granules.^12^ Although potential toxicity mechanisms of hIAPP oligomers have been suggested, the mechanism of hIAPP oligomer formation remains elusive.

Divalent metal ions [*e.g.*, Cu(ii), Zn(ii)] have been accepted as a major cause of the formation of hIAPP oligomers, in that both metal ions may suppress hIAPP fibrillation and form stable oligomers.^7^,^13^–^16^ Since the interaction site of Cu(ii) or Zn(ii) on hIAPP has been identically proposed to His18,^14^,^17^ cooperative and competitive effects of these metal ions are also expected as observed in amyloid-β.^18^ Cu(ii) and Zn(ii) also play an important role in the pathology of T2DM. Alteration in Cu(ii) metabolism has been related to T2DM based on several clinical reports demonstrating an elevated level of Cu(ii) in the serum and plasma of patients with diabetes mellitus.^19^–^25^ In particular, the serum Cu(ii) level is associated with glycemic control in T2DM patients.^26^ One *in vivo* study indicated that gastrointestinal copper absorption is enhanced in diabetic rats.^27^ It is, however, still unclear whether the Cu(ii) level is elevated in pancreatic cells of T2DM patients. Considering the fact that insulin exocytosis occurs near blood vessels,^28^ an increased Cu(ii) level in serum would facilitate interactions between Cu(ii) and hIAPP, which is co-secreted with insulin.^29^ In addition, other studies suggest that the interaction of hIAPP with Cu(ii) could induce the production of toxic aggregates and ROS.^13^ Zn(ii) could be involved in the stabilization and release of insulin in the β-cells of the pancreas,^30^ the organ with the highest level of this metal ion; indeed, Zn(ii) is found at millimolar concentration in β-cell granules.^31^ Taken together, the interactions between hIAPP and these divalent ions seem to be closely linked to the formation of toxic peptide aggregates.

Herein, we report the structures of metal-free and Cu(ii)-/Zn(ii)-associated hIAPP oligomers, as well as mechanistic details of their assembly using multiscale biophysical approaches, including circular dichroism (CD) spectroscopy, transmission electron microscopy (TEM), electrospray ionization-ion mobility-mass spectrometry (ESI-IM-MS), gel electrophoresis with Western blot, small-angle X-ray scattering (SAXS), and molecular dynamics (MD) simulations. Overall, the results from our investigations of metal-free and metal-associated hIAPP oligomerization present the structural features and assembly mechanisms of early-stage amyloid oligomers, advancing our understanding of how hIAPP–metal interactions could direct peptide assembly that is relevant to toxicity.

## Results and discussion

### Off-pathway oligomerization of hIAPP mediated by Cu(ii)

We first investigated hIAPP fibrillation in the absence and presence of Cu(ii) and Zn(ii) using CD spectroscopy (Fig. 1b). The secondary structure of metal-associated hIAPP and metal-free hIAPP was dominantly random coil at the initial stage. After 6 h incubation, suppression of hIAPP fibrillation in the presence of Cu(ii) was observed under our experimental conditions (20 mM HEPES, pH 7.5, 20 mM NaCl; 37 °C; constant agitation at 300 rpm). The secondary structure of hIAPP remained as random coil in the presence of Cu(ii), whereas high β-sheet propensity was observed in Zn(ii)-treated and metal-free hIAPP. We also examined the morphology of the 6 h-incubated hIAPP aggregates using TEM with 80 keV electron beam energy (Fig. 1c). The morphology of Cu(ii)-associated hIAPP aggregates was small and rounded, distinct from that of amyloid fibrils. This is consistent with the previous studies which reported the Cu(ii)-mediated formation of hIAPP aggregates with only a few tens of nanometers in diameter.^7^,^13^ In contrast to the Cu(ii)-treated form, Zn(ii)-treated and metal-free hIAPP assembled into fibrils after 6 h of incubation (Fig. 1c). We further examined the morphology of hIAPP aggregates using high-resolution TEM (HR-TEM) with 200 keV electron beam energy to obtain more detailed images of hIAPP aggregates. Oligomeric species were commonly found in all of the hIAPP samples after 3 h of incubation (denoted with yellow boxes in Fig. 1d). However, the production of the fibrillar aggregates was suppressed by the addition of Cu(ii), whereas the oligomeric species in Zn(ii)-treated and metal-free conditions were converted to fibrillar aggregates after 6 h incubation. These results imply that Cu(ii) mediates the generation of off-pathway hIAPP oligomers and maintains the random coil structure of hIAPP.

**Fig. 2:**
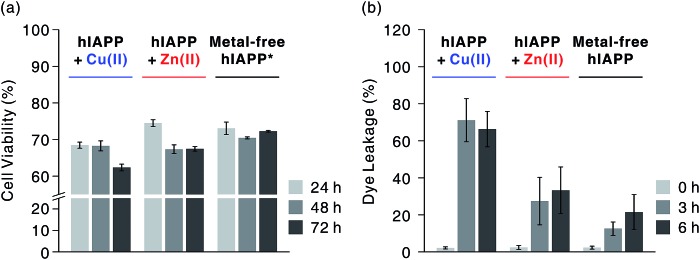
Cytotoxicity investigations of metal-free and metal-mediated hIAPP oligomers. (a) Toxicity of hIAPP preincubated with and without metal ions for 3 h in rat INS-1 pancreatic β-cells, observed over 24–72 h. Conditions: [hIAPP] = 10 μM; [CuCl_2_ or ZnCl_2_] = 20 μM. Cell viability was normalized with the background for each condition (*e.g.* toxicity of Cu(ii)-treated hIAPP was normalized with the toxicity of Cu(ii)). Cell viability for Cu(ii) and Zn(ii) is suggested in Fig. S1.† *According to inductively coupled plasma mass spectrometry analysis, the medium used for cell culture includes *ca.* 0.6 and 6.5 μM of Cu and Zn, respectively, and the INS-1 cell lysates includes *ca.* 0.7 μM and 1.8 μM of Zn, respectively. This indicates that metal-associated hIAPP could exist even in our metal-free condition, inducing toxicity in living cells. (b) Dye leakage test for hIAPP with or without pre-incubation for 3 and 6 h. Conditions: [hIAPP] : [vesicles] = 1 : 100 ([hIAPP] = 2 μM; vesicles are composed of POPS and POPC at a ratio of 7 : 3).

The highly toxic effects of Cu(ii)-associated hIAPP oligomers have been previously reported.^7^,^13^ Chen and co-workers reported that Cu(ii) increased the cytotoxicity of hIAPP aggregates by facilitating apoptosis-promoting effects.^7^ In particular, they suggest that Cu(ii)-triggered ROS could disrupt mitochondria and then initiate apoptosis in pancreatic cells. We conducted a toxicity study using rat insulinoma-1 (INS-1) pancreatic β-cells. The results of this experiment also support the toxicity of Cu(ii)-associated hIAPP oligomers. Higher toxicity of Cu(ii)-associated hIAPP oligomers, generated by preincubation of Cu(ii) and hIAPP, was observed compared to both metal-free hIAPP and Zn(ii)-associated hIAPP (Fig. 2a). When INS-1 pancreatic β-cells were treated with Cu(ii)-associated hIAPP oligomers and incubated for different durations (24–72 h), their cytotoxicity increased in a time-dependent manner. Additionally, a dye leakage assay was also carried out to verify the membrane-disruption effects of Cu(ii)-associated hIAPP oligomers. Previous studies suggest that hIAPP oligomers could induce membrane disruption in a manner similar to antimicrobial peptides.^8^,^32^,^33^ As shown in Fig. 2b, hIAPP preincubated with Cu(ii) for 3 h was observed to impair membrane integrity by *ca.* 70%, whereas metal-free or Zn(ii)-associated hIAPP aggregates triggered only 10–30% dye leakage. Even after 6 h of incubation, Cu(ii)–hIAPP presented higher dye leakage effects (*ca.* 65%) compared to metal-free and Zn(ii)-treated hIAPP. Thus, our results suggest that when Cu(ii) is present, hIAPP monomers are redirected to form off-pathway aggregates which exert increased toxicity towards pancreatic β-cells and cause deterioration of membrane integrity.

### Structural analyses of metal-free and metal-associated hIAPP monomer

**Fig. 3:**
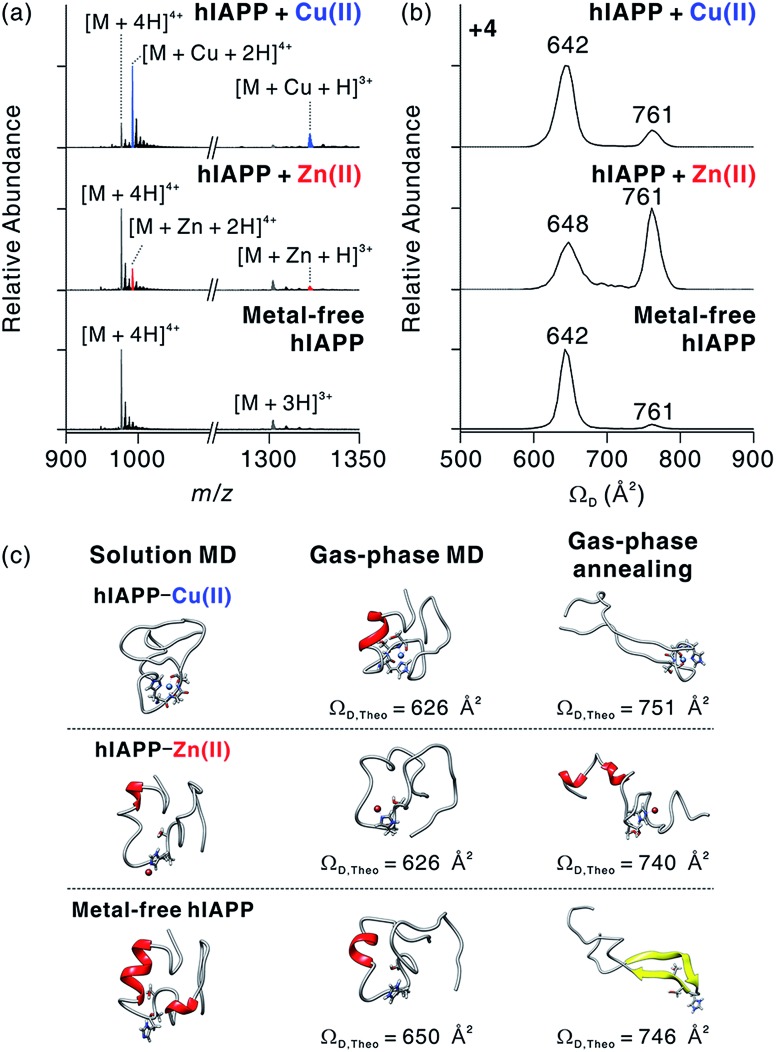
Molecular interactions and conformational changes in metal-associated hIAPP. (a) ESI-MS spectra for monomeric hIAPP with and without metal ions. Conditions: [hIAPP] = 5 μM; [CuCl_2_ or ZnCl_2_] = 10 μM; 100 mM ammonium acetate. The average mass for hIAPP is indicated as *M*. (b) IM-MS spectra for +4-charged metal-associated and metal-free hIAPP. (c) Representative MD-simulated structures of hIAPP monomer in solution and the gas phase.

To understand the structural features of hIAPP monomers, we used ESI-IM-MS that provides collision cross-section (*Ω*_D_) and the mass-to-charge ratio (*m*/*z*) of ions. ESI-IM-MS has been used to probe individual conformers of intrinsically disordered proteins (IDPs), and to determine their assembly dynamics based on variations of *Ω*_D_ in the gas phase.^34^–^36^ ESI-IM-MS has also been employed to understand the conformational dynamics of metal-free hIAPP and its assemblies.^37^,^38^ We first probed the complex formation between hIAPP and metal ions upon the treatment with metal ions (Fig. 3a). The mass spectra of hIAPP without metal ions dominantly exhibit a 4+ charged state. When Cu(ii) and Zn(ii) ions were added to hIAPP solution, 1 : 1 metal–protein complexes were observed with similar charge state distributions. In the case of Cu(ii), the holo form ([M + Cu + 2H]^4+^; *m*/*z* 992.2) shows higher abundance than the apo form ([M + 4H]^4+^; *m*/*z* 976.8). On the other hand, for Zn(ii), the apo form was higher in abundance than the holo form ([M + Zn + 2H]^4+^; *m*/*z* 992.7). This result is likely to reflect the higher affinity of Cu(ii) toward hIAPP than that of Zn(ii) toward hIAPP (for Cu(ii), *K*_a_, *ca.* 8.9 × 10^7^ M^–1^; for Zn(ii), *K*_a_, *ca.* 9.1 × 10^5^ M^–1^; Fig. S2†).

The binding sites of Cu(ii) and Zn(ii) on hIAPP were identified using electron capture dissociation (ECD).^39^,^40^ ECD analysis indicates that the metal ions are located in the central region of hIAPP that includes His18 (Fig. S3†). This result is consistent with previously reported binding sites for Cu(ii)^13^,^17^ and Zn(ii).^14^ We further tested the role of His18 in metal binding by varying the pH of hIAPP solution from 7 to 5. The His residue in a protein is protonated at pH 5 (p*K*_a_ ∼ 6.1), and the protonation inhibits the interaction between His and metal ions.^14^ As expected, the formation of hIAPP–metal complexes dramatically decreases at pH 5 (Fig. S4†). These results further support that the molecular interactions between hIAPP and metal ions occur at His18 of hIAPP.

We also investigated the conformational features of metal-associated and metal-free hIAPP monomers (Fig. 3b). Ion mobility spectra of +4-charged hIAPP ions are plotted with calibrated *Ω*_D_ values on the *x* axis (Fig. S5†). The distributions indicate that two major conformers of hIAPP [*i.e.*, the compact (*Ω*_D_ = 642–648 Å^2^) and extended (*Ω*_D_ = 761 Å^2^) conformers] were present in both metal-associated and metal-free states. The relative abundance of the compact conformer in Cu(ii)-bound and metal-free hIAPP ions is higher than that of the extended conformer. In contrast, for Zn(ii)-bound hIAPP ions, the abundance of the extended conformer is higher than that of the compact conformer. Notably, hIAPP associated with various other metal ions (Ca^2+^, Mg^2+^, and 2Na^+^) also preferred the extended conformation to the compact conformation as indicated by the results of ESI-IM-MS (Fig. S6†). Previously, Bowers and co-workers suggested that electrostatic repulsion between charged residues could stabilize the extended conformer of metal-free hIAPP in the gas phase.^36^ We also infer that the extended conformer of metal-associated hIAPP ions is stabilized by electrostatic repulsion during the transfer of ions from solution to the gas phase.

The prevalence of compact conformers for Cu(ii)-associated hIAPP ions is likely to originate from its distinct coordination property. The previous EPR study on hIAPP–Cu(ii) suggested that Cu(ii) could be coordinated to hIAPP in a 3N1O form with two deprotonated nitrogens in the amide backbone of Ser19 and Ser20, a nitrogen donor atom in the His18 residue, and an oxygen donor atom of Ser20.^17^ Such residues composed of two negatively charged and two polar atoms stabilize the positive charge of Cu(ii). Thus, it is expected that the coordinated Cu(ii) cannot exert sufficient electrostatic repulsion to alter the structures of hIAPP in the gas phase.

To understand the role of metal ions in hIAPP structures, we performed molecular dynamics (MD) simulations in both solution and the gas phase. Replica exchange MD (REMD) simulations of hIAPP monomer in implicit solvent were firstly performed for effective sampling of metal-free hIAPP conformation in solution. Structures with a residual α-helix (Fig. 3c) were dominant in the structural distribution of hIAPP (Fig. S7†), in accordance with a previous study.^36^ Another structural family with a residual β-strand^36^ was also observed at a lower abundance (Table S1†). MD simulations of these structures were further performed in the gas phase (Fig. S8†). The simulations yielded the structures with theoretical *Ω*_D_ corresponding to experimental *Ω*_D_ of the compact conformer. This result implies that the compact conformer has most likely originated from the structures in solution. We further conducted simulated annealing in the gas phase to generate and understand candidate structures of the extended conformer. We chose the lowest-energy structures which have theoretical *Ω*_D_ values that agree (±3%) with the experimental *Ω*_D_ value of the extended conformer (Fig. S9†). The most stable conformation for the extended conformer is the extended β-strand structure with the N-terminal and C-terminal regions aligned in an anti-parallel orientation.

Based on the binding sites deduced from ECD-MS and literature reports,^13^,^14^,^17^ metal-associated hIAPP was also simulated using MD. We ligated a metal ion to the most abundant structures in metal-free hIAPP and simulated the peptide in an explicit solvent. Compared to metal-free hIAPP, metal-associated hIAPP monomers display similar structures, but metal-hIAPP coordination at His18 causes a local change around His18 (Fig. 3c). MD simulations of metal–hIAPP complexes in the gas phase generate compact ions with small *Ω*_D_, suggesting that the compact conformers observed in IM-MS were transferred from the structure in solution. Analogously to metal-free hIAPP ions, it was necessary to sample candidate structures of gas-phase hiAPP–metal complex ions using simulated annealing (Fig. S10 and S11†). In the extended conformer of the hIAPP–Zn(ii) complex, the local structure around His18 was rearranged to stabilize Zn(ii). In the case of Cu(ii), while hIAPP–Cu(ii) coordination maintains the local structure around His18, carbonyl groups in the backbone of the C-terminal region stabilize the charged Lys1 and N-terminus, similar to the metal-free hIAPP ions.

The structural difference in the extended conformer has its origin in the 3N1O coordination of hIAPP–Cu(ii) complexes. The maximum charge of hIAPP–Cu(ii) complexes is +3 in solution (N-terminus, Lys1, and Arg11). Even if one of the two deprotonated nitrogens in amide backbone is reprotonated to yield +4 ion in the gas phase, the Cu(ii)-coordinated region of hIAPP would remain as a +1 charged state, which is analogous to the protonated His18 in metal-free hIAPP. In contrast, for the hIAPP–Zn(ii) ion, only two sites from among the possible protonation sites should be protonated to yield the +4 ion. Consequently, the distribution of positive charges in hIAPP monomer causes the structural differences in the extended conformer. To summarize, the molecular interaction between Cu(ii) and hIAPP causes the regional localized change in hIAPP structure in solution and the gas phase.

### Assembly mechanisms of small-sized hIAPP oligomers in metal-associated and metal-free states

**Fig. 4:**
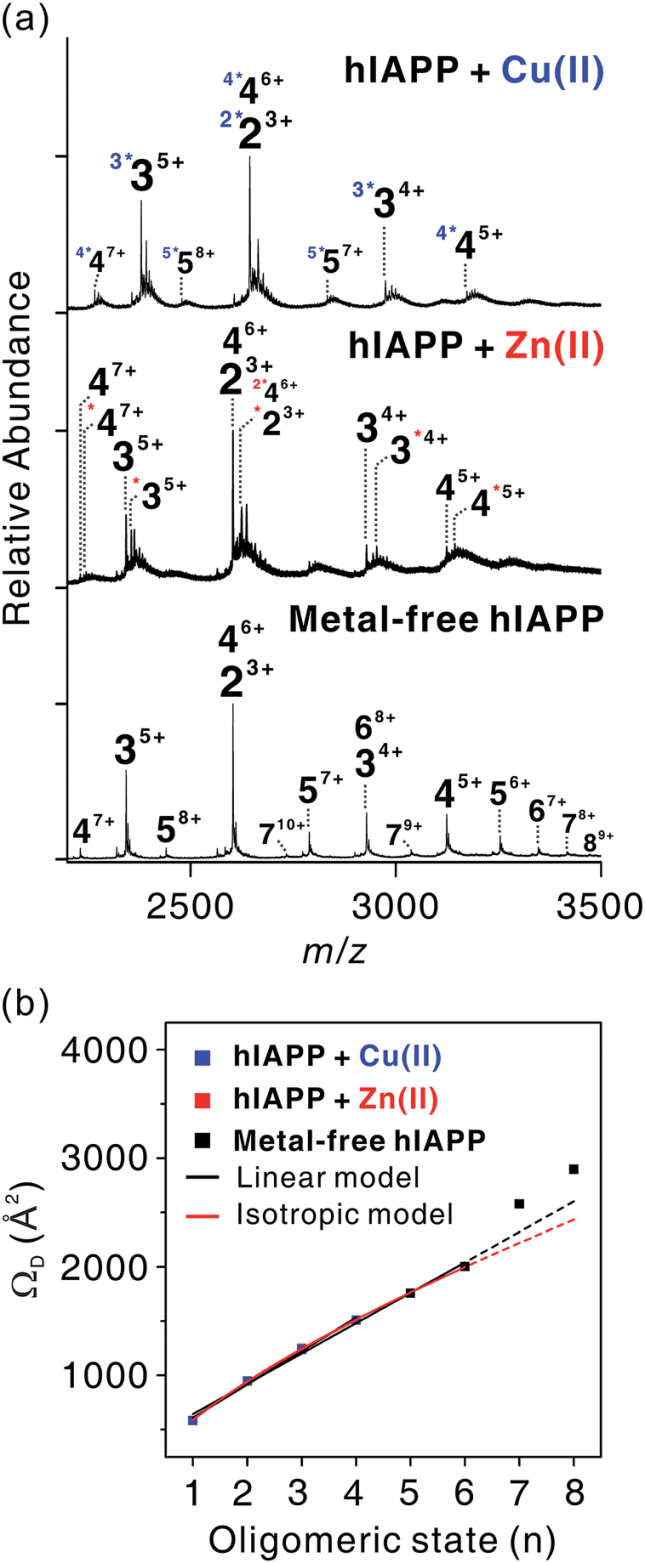
Assembly mechanism of hIAPP oligomers. (a) ESI-MS for oligomeric hIAPP incubated with and without metal ions. *z*-Charged *n*-mer ion is denoted as *n*^*z*+^, and left superscript with asterisks *k* (^k^*) indicates *k* metal ions included in the oligomers. (b) Correlations between oligomeric state (*n*) and *Ω*_D_ of *n*^*n*+1^ ions for experimentally detected oligomers. Calibration curve of the oligomers is available in Fig. S13.†

We investigated the oligomerization mechanisms of metal-associated and metal-free hIAPP incubated in solution using ESI-IM-MS. The mass spectra of incubated hIAPP show that metal-associated and metal-free hIAPP oligomers form up to tetrameric and octameric states, respectively (Fig. 4a). Although broad peaks corresponding to pentameric and hexameric states of hIAPP were also observed in ESI-MS of metal-treated hIAPP, we excluded them from the identified peak lists due to the low abundance of metal-specific peaks. Then we only treated metal-specific peaks with the high abundance (Fig. S12†). The *n*-mer hIAPP ions with *n* + 1 charge state were dominantly observed in both metal-associated and metal-free state. Other charge states (*n* + 2 and *n* + 3) of hIAPP *n*-mer were also observed at lower abundance. Cu(ii)-associated hIAPP oligomers mainly exhibit 1 : 1 metal-to-peptide stoichiometry, whereas a single Zn(ii) ion binds to hIAPP oligomers (Fig. S12†). As shown in the monomeric state, the 1 : 1 metal-to-peptide ratio in Cu(ii)-associated hIAPP oligomer implies that Cu(ii) maintains its high affinity toward each monomer within the oligomer. For Zn(ii), it is expected that multiple interactions of a single Zn(ii) ion with hIAPP induce oligomer assembly, as suggested in a previous study.^16^

Experimental *Ω*_D_ values for *n*-mer hIAPP with *n* + 1 charge state were plotted against *n* in Fig. 4b. The *Ω*_D_ values of metal-free oligomers generally agree with those of metal-free oligomers previously reported.^38^ Up to the hexameric state of metal-free hIAPP, their *Ω*_D_ values can be fitted to a linear function and a two-thirds power function (Table S2†). The linear model indicates that monomers are aligned in the one direction.^41^ The two-third power function indicates that monomers are isotropically assembled to globular conformations.^41^ Previous studies using ESI-IM-MS suggest that metal-free hIAPP are linearly^38^ or isotropically^42^ assembled. Our fitting results show that the isotropic growth is slightly better matched to the experimental *Ω*_D_ values. Both models could be possible scenarios for hIAPP oligomerization, however.

The *Ω*_D_ values of both metal-associated and metal-free oligomers show a similar trend up to the tetramer stage. It is noteworthy that a minor change in the conformation of Cu(ii)-associated dimer was observed in *n* + 3 charge state (Fig. S14–S17†). In the dimer with +3 charge state, four distinct peaks were observed in the arrival time distributions (Fig. S15†). The peak at the center shows the *Ω*_D_ value similar to that of the metal-free dimer, while *Ω*_D_ values of the other peaks indicate the presence of small and large conformers. This implies that the overall sizes of the oligomer ions are similar at low charge states in the gas phase, but a minor change is observable at high charge states. Such a loss of conformational properties in metal-associated oligomers can be attributed to the gas-phase collapse at low charge state.^43^

In metal-free oligomers, a discrete trend from the linear and isotropic models is observed in heptamer and octamer forms. This may be explained by a transition from the isotropic or the linear assembly model to another growth model. An earlier study suggests that there is a transition from the isotropic to the linear growth during the self-assembly of peptides.^41^ Although metal-containing oligomers larger than pentamers were not observed, it is expected that metal ions would induce a conformational change as the size of the oligomer increases.

In summary, during the assembly of small-sized oligomers, metal-associated and metal-free oligomers form through a similar growth model (linear or isotropic) up to tetramer stage. After the tetramer state, however, the molecular interactions between metal ions and hIAPP may divert the assembly process to a different pathway from that of the metal-free state.

### Characterization of large-sized oligomers for metal-associated and metal-free hIAPP

**Fig. 5:**
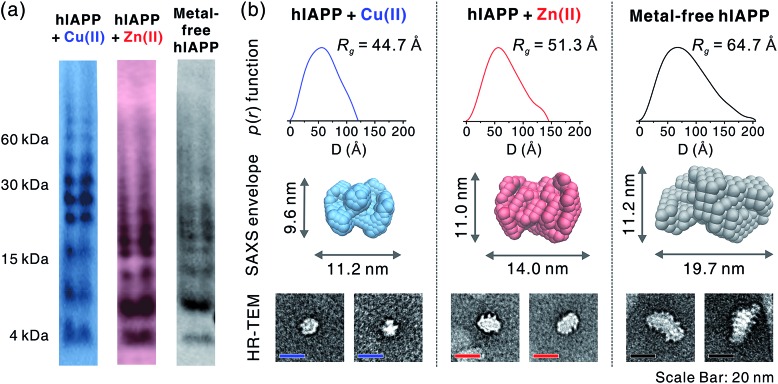
Size analyses of hIAPP oligomers. (a) Oligomerization monitored using gel electrophoresis with Western blotting (amylin antibody H-50). (b) Solution SAXS analysis of metal-mediated and metal-free oligomers. *p*(*r*) from averaged scattering patterns of each oligomer solution is suggested with *R*_g_ values (up). SAXS envelope structures reconstructed from *p*(*r*) (mid) are suggested with the representative TEM image of the individual oligomers (down).

To gain a better understanding of metal-mediated hIAPP assemblies from small-sized oligomers to large-sized oligomers, gel electrophoresis with Western blotting (gel/Western blot) using a H-50 antibody was performed for hIAPP samples incubated with and without Cu(ii) or Zn(ii). To obtain the soluble oligomer fraction, we prepared the supernatant from the incubated solution using centrifugation. The gel/Western blot analysis of the supernatant, showed a wide distribution of bands ranging from 4 to 60 kDa (1–15 mers) in both metal-associated and metal free hIAPP (Fig. 5a). Cu(ii)-treated hIAPP showed distinct bands around 30 kDa, whereas Zn(ii)-treated and metal-free hIAPP displayed bands around 15 kDa. This result implies that the molecular interaction of hIAPP with Cu(ii) could induce the formation of soluble oligomers with different assembly states compared to Zn(ii)-treated and metal-free states.

The supernatant of the incubated hIAPP solution was further analyzed using solution SAXS. We obtained scattering patterns of the soluble fraction of the solution incubated with and without metal ions (Fig. S18a†). Then, the radius of gyration (*R*_g_) was estimated from Guinier fit analysis (Fig. S18b†). Based on the *R*_g_ values and scattering patterns, the pair distance distribution function (*p*(*r*)) was generated using program GNOM^44^ to obtain a three-dimensional envelope structure^45^ of hIAPP oligomers in the supernatant (Fig. 5b). The *R*_g_ values of each oligomer increase in the following order: Cu(ii) (44.7 Å) < Zn(ii) (51.3 Å) < metal-free (64.7 Å). The maximum distances (*D*_max_) in *p*(*r*) and the dimensions of SAXS envelopes also increase in the same order as *R*_g_. SAXS envelopes of metal-containing and metal-free oligomers were reconstructed from the *p*(*r*) of the oligomers.

Although SAXS envelopes were deduced from the ensemble measurement of diverse oligomeric states contained in the sample, we assumed that SAXS scattering patterns would reflect the size of the abundant species in the supernatant. We attempted to directly compare the envelopes with magnified TEM images of hIAPP oligomers. When compared with TEM images, SAXS envelopes show a good agreement with negatively-stained oligomers. This result supports the concept that SAXS envelopes partially represent the individual oligomers in the TEM images despite the averaging of structural information (Fig. 5b), as suggested in the previous study of amyloid oligomers.^46^

While Cu(ii)-associated hIAPP shows a distinct band around 30 kDa by gel/Western blot, it exhibits the smallest *R*_g_ value in SAXS measurement. In contrast, Zn(ii)-associated hIAPP and metal-free hIAPP show oligomers of approximately 15 kDa in gel/Western blot, but *R*_g_ values of Zn(ii)-treated and metal-free hIAPP are greater than those of Cu(ii)-treated hIAPP. We suppose that Cu(ii) stabilizes the assembly state of hIAPP into an intermediate state between small oligomeric state and fibrillar aggregates. On the other hand, such intermediate species are not found in the gel/Western blot and SAXS of Zn(ii)-associated and metal-free oligomers which are readily converted into fibrillar aggregates.

### Distinct features in the oligomerization of Cu(ii)-associated hIAPP

In our studies, Cu(ii)-associated hIAPP formed off-pathway aggregates that were stabilized as soluble oligomers. We first confirmed Cu(ii)-associated oligomers by TEM (Fig. 1c and d). SAXS envelope obtained from the Cu(ii)-treated hIAPP solution further supports the presence of the soluble oligomers similar to those in the TEM images (Fig. 4b). These oligomeric species showed relatively high toxicity in pancreatic β-cells, compared to Zn(ii)-containing and metal-free forms (Fig. 2a). The disruptive effect of Cu(II)-containing hIAPP on the membrane of lipid vesicles was especially significant (Fig. 2b).

Fibrillation of hIAPP occurs through the sequential process of (i) formation of an α-helical intermediate,^47^ (ii) β-turn formation at the central region of hIAPP,^48^ and (iii) the assembly of a β-sheet rich fibril.^49^ Protonation or Zn(ii) coordination at His18 of the central region of the polypeptide has an inhibitory effect on the process of the intermediate formation due to following reasons. First, electrostatic repulsion by protonation at His residues disrupts protein–protein interaction of hIAPP.^50^ Second, Zn(ii) coordination stabilizes the structure of off-pathway intermediates.^16^ The 3N1O coordination of Cu(ii) around His18 also restricts the conformational change of the hIAPP structure.^17^ Our ESI-IMS-MS results and MD simulations of hIAPP monomer (Fig. 3) also suggest that its coordination to Cu(ii) with the relatively high stability of hIAPP–Cu(ii) complexes could restrict the change of the regional structure of hIAPP monomer in both solution and the gas phase. Moreover, the stoichiometry of 1 : 1 metal to peptide in Cu(ii)-containing oligomers (Fig. 4a) implies that the interaction with Cu(ii) is still present in the oligomeric states of hIAPP.

We infer that such molecular interactions between hIAPP and Cu(ii) are crucial for the formation of off-pathway aggregates and their toxicity. CD spectra and TEM images (Fig. 1) showed that Cu(ii)-associated oligomers would have a random coil propensity rather than a β-sheet propensity. These facts imply that while hIAPP–Cu(ii) interaction suppresses the formation of hIAPP fibrils, the hydrophobic regions in hIAPP can interact with each other *via* non-specific intermolecular hydrophobic interactions. Most of the hIAPP sequences except for a few residues in the N-terminal region are composed of highly hydrophobic residues that participate in β-sheet alignment of hIAPP fibrils. These hydrophobic regions of hIAPP in Cu(ii)-containing oligomers may have random conformations rather than aligned structures. In general, β-sheet aligned fibrils have less toxic and deleterious effects on membranes than soluble amyloid oligomers with more exposed hydrophobic regions.^2^,^8^,^32^ Thus, the relatively high toxicity of Cu(ii)-mediated oligomers seems to be correlated with the exposure of hydrophobic residues in a random coil state. As a result, Cu(ii) binding to hIAPP with relatively high affinity (*K*_a_, *ca.* 10^8^ M^–1^) could cause the self-assembly of hIAPP with random coil structures, thereby inducing the formation of toxic aggregates.

## Conclusions

The oligomerization process of metal-treated and metal-free hIAPP has been characterized using multiscale biophysical techniques. The structural characteristics of metal-associated and metal-free hIAPP oligomers were examined using CD, TEM, and SAXS. We confirmed the toxicity of these oligomers using the MTT assay and dye leakage assay. The molecular interactions of metal-containing hIAPP, and the growth mechanisms of the small-sized oligomers were investigated using ESI-IM-MS and MD simulations. Based on the results obtained with these multiple biophysical methods, we suggest that the molecular interaction between hIAPP and Cu(ii) stabilizes the secondary structure of the oligomers into random coil, thereby inducing the formation of toxic and off-pathway aggregates. Our findings with mechanistic details on toxic assemblies of Cu(ii)-containing hIAPP may provide insights into developing a rational strategy for the design of effective therapeutic agents (*e.g.*, metal chelating agents^51^,^52^). Moreover, our approaches presented herein can be generally applied to identify the structural properties and oligomeric assemblies of other amyloidogenic proteins.^46^,^53^

## Supplementary Material

Supplementary information
