# Supplementary material for: Structure and assembly mechanisms of toxic human islet amyloid polypeptide oligomers associated with copper
Source: Chem Sci. 2016 May 16;7(8):5398–406. doi: 10.1039/c6sc00153j (PMC6020819; doi:10.1039/c6sc00153j)
Supplement: Supplementary file 1 [file SC-007-C6SC00153J-s001.pdf]

Electronic Supplementary Material (ESI) for Chemical Science.
This journal is © The Royal Society of Chemistry 2016

SUPPLEMENTARY INFORMATION

for

Structure and assembly mechanisms of toxic human islet amyloid polypeptide oligomers associated with copper

Shin Jung C. Lee‡,[ab] Tae Su Choi‡,[bc] Jong Wha Lee‡,[bc] Hyuck Jin Lee,[a] Dong-Gi Mun,[b] Satoko Akashi,[d] Sang-Won Lee,[c] Mi Hee Lim,*[a] and Hugh I. Kim*[c]

[a]Department of Chemistry, Ulsan National Institute of Science and Technology (UNIST), Ulsan 44919, Republic of Korea

[b]Department of Chemistry, Pohang University of Science and Technology (POSTECH), Pohang 37673, Republic of Korea

[c]Department of Chemistry, Korea University, Seoul 02841, Republic of Korea

[d]Graduate School of Medical Life Science, Yokohama City University, Yokohama, Kanagawa 230-0045, Japan

‡ These authors equally contributed to this work

*To whom correspondence should be addressed: hughkim@korea.ac.kr and mhlim@unist.ac.kr

METHODS

Sample preparation. Human islet amyloid polypeptide (hIAPP) was synthesized by Peptron (Daejeon, Republic of Korea) with a purity of 95%, and used without further purification. To remove preformed aggregates, hIAPP was dissolved in 1,1,1,3,3,3-hexafluoro-2-propanol (HFIP, purchased from Tokyo Chemical Industries Inc., Ltd., Tokyo, Japan) at a concentration of 100 μM. After 2 h treatment with HFIP at the room temperature, HFIP was evaporated from the hIAPP solutions. For further removal of preformed aggregates, the obtained protein film was re-dissolved to 100 μM in HFIP, and the resulting solution was re-evaporated under the vacuum.

Circular dichroism (CD) spectroscopy. The secondary structures of hIAPP were probed using Jasco J-815 circular dichroism spectrometer (Tokyo, Japan). The concentrations of hIAPP and metal chloride salts used for experiments were 10 μM and 70 μM, respectively. CD spectra were obtained from 190 nm to 260 nm with a scan speed of 20 nm/min [a quartz cuvette of 0.1 cm path length (Starna, Atascadero, CA, USA)]. Averaged spectra obtained from three independent experiments were used for analysis.

Transmission electron microscopy (TEM). TEM samples were prepared using 400-mesh formvar/carbon-coated copper grids (Electron Microscopy Science, Hatfield, PA, USA). 5 μL aliquot of 3 h or 6 h incubated hIAPP (20 μM in 20 mM HEPES, pH 7.5, 20 mM NaCl) was applied to a grid for 3 min followed by removal. For negative staining, the grid was incubated with 0.5% (w/v) uranyl acetate solutions for 1 min. After incubation, remaining uranyl acetate on the grid was removed using a micropipette. TEM and HR-TEM images were taken with a JEM-

1011 transmission electron microscope (JEOL, Tokyo, Japan) at POSTECH Biotech Center (Pohang , Republic of Korea) and JEM-2200FS with image CS-corrector (JEOL, Tokyo, Japan) at POSTECH National Institute for Nanotechnology (Pohang, Republic of Korea), respectively.

Cytotoxicity studies. Cell viability was measured using the MTT assay [MTT = 3-(4,5-dimethylthiazol-2-yl)-2,5-diphenyltetrazolium bromide]. The rat insulinoma-1 (INS-1) pancreatic β-cells, purchased from AddexBio (San Diego, CA), were treated with hIAPP (10 μM in 20 mM HEPES, pH 7.5, 20 mM NaCl) that was preincubated for 3 h with constant agitation (250 rpm) at 37 °C and then incubated over 24–72 h under 5% CO_2 according to the previous report.[2] After incubation, MTT (25 μL of 5 mg/mL in phosphate buffered saline, pH 7.4, GIBCO) was added to the cells. Formazan produced by the cells was solubilized using an acidic solution of *N*,*N*-dimethylformamide (DMF, 50% v/v, *aq*) and sodium dodecyl sulfate (SDS, 20% w/v) overnight at room temperature in the dark. The absorbance was measured at 600 nm using a SpectraMax M5e microplate reader (Molecular Device, Sunnyvale, CA, USA). Cell viability was calculated by normalizing the absorbnace of hIAPP-treated cells to the absorbance of cells containing background solution.

Dye leakage assay. To assess the membrane disruptive effects of oligomeric states, dye leakage tests were conducted. Large unilamellar vesicles were prepared according to the procedure previously published.[3] 5(6)-carboxyfluorescein (20 mM) and mixed lipids (POPS : POPC = 7 : 3; 10 mM) were hydrated and went through freeze-thaw cycles (repeated more than 20 times). Then, the lipid solution was introduced to an extruder (Avanti Polar Lipids, Inc., Alabaster, AL, USA) equipped with a polycarbonate membrane (100 nm pore size). Through a Sephadex G-10 column,

dye-encapsulating vesicles were separated from free dyes. hIAPP and metal chloride salts were prepared at the concentration of 20 μM and 140 μM, respectively, and the fluorescence was measured with 10-fold diluted hIAPP and dye-encapsulating vesicles ([hIAPP] = 2 μM and [vesicles] = 200 μM). The magnitude of dye leakage was determined according to the following equation (S.1).

$$\text{Dye Leakage (\%)} = \frac{F - F_0}{F_{100} - F_0} \qquad \text{(S.1)}$$

F_{100} indicates the fluorescence intensity under 100% leakage attained by the addition of 0.1% Triton X-100. F_0 means the baseline fluorescence, and F is the fluorescence intensity that was measured 2 min after the addition of hIAPP to dye-trapping vesicles.

Nanoelectrospray ionization ion mobility-mass spectrometry (nESI-IM-MS). Ion mobility-mass spectrometry (IM-MS) for monomeric hIAPP was performed using Synapt G2 HDMS traveling wave ion mobility orthogonal acceleration time-of-flight mass spectrometer equipped with an on-line nanoelectrospray ionization source (Waters, Manchester, UK) at the POSTECH chemistry building (Pohang, Republic of Korea). The capillary voltage was adjusted to 3.5 kV, and the source temperature was set to 100 °C. 10 μM of hIAPP without or with 20 μM metal chloride salts in 100 mM ammonium acetate (pH 7.5) was freshly prepared and sprayed out directly. Ion mobility wave height and wave velocity were adjusted to 7 V and 200 m/s. For the detection of oligomeric states of hIAPP, samples were prepared with the same protocol as the SAXS experiments except that 100 mM ammonium acetate was used instead of Tris-HCl buffer. Ion mobility for oligomeric hIAPP was measured with the Waters Synapt G2 HDMS mass spectrometer equipped with an off-line nanoelectrospray ionization emitter at Yokohama City University (Yokohama, Japan). HUMANIX nanospray tips with 8 μm internal diameter

(Hiroshima, Japan) were used for the measurement. The capillary voltage and source temperature were set to 1 kV and 60 ºC. Ion mobility wave height and velocity were adjusted to 40 V and 600 m/s. Drift time was calibrated to collision cross sections following a previously developed method (Fig. S4 and S12).[1]

Fluorescence dye titration for metal binding constant. Binding affinities of metal ions to hIAPP were assessed by fluorescence dye titration methods using metal-specific fluorescent dyes. To confirm reproducibility of this method, we repeatedly conducted the experiments with multiple dyes. For the measurement for Zn(II) binding of hIAPP, FluoZin-3 and FluoZin-1 were employed. FluoZin-3 and Newport-Green were used for the estimation of the binding affinity between Cu(II) and hIAPP. Freshly prepared hIAPP was added to the solution containing a metal chloride salt (5 μM) and metal-specific dyes (5 μM). Metal binding affinity of hIAPP was deduced by the following equations (S.2) and (S.3). The binding constants between metal ions and fluorophores were determined by the manufacturer (Life Technologies, Carlsbad, CA).

$$I_M = I_0 \cdot \frac{[dye]_o}{[dye]_{tot}} + I_{100} \cdot \frac{[dye-metal]_{eq}}{[dye]_{tot}} \tag{S.2}$$

$$K_a = \frac{[hIAPP-metal]_{eq}}{[hIAPP]_{eq} \cdot [metal]_{eq}} = \frac{([metal]_{tot} - [dye-metal]_{eq} - [metal]_{eq})}{([hIAPP]_{tot} - ([metal]_{tot} - [dye-metal]_{eq} - [metal]_{eq})) \cdot [metal]_{eq}} \tag{S.3}$$

I_M is the fluorescence intensity of the sample solution; I_0 indicates the baseline fluorescence intensity of metal-specific dyes. I_{100} corresponds the fluorescence when total binding of metal-specific dyes with metal ions is accomplished.

Electron capture dissociation (ECD) mass spectrometry. ECD-MS experiments were performed using a 7-tesla FT-ICR mass spectrometer (LTQ-FT Ultra, Thermo Electron, San Jose,

CA, USA). hIAPP (2 μM) without and with 10 μM metal chloride salts were directly injected to mass spectrometer using a syringe pump (model 781100, KD Scientific) at a flow rate of 18 μL/min. Electrospray ionization (ESI) was performed through a laboratory-built nanoESI source. The ESI voltage and capillary temperature were set to 3.5 kV and 200 °C, respectively. Precursor ions were isolated in a linear ion trap with an isolation width of 3 Th and fragmented with ECD in the ICR cell. Collision energy of 5 eV and the duration time of 60 ms were used. Automatic gain control (AGC) target value of ECD was set to 5×10^5. Mass spectra were obtained at a resolution of 100,000.

Synchrotron small-angle X-ray scattering (SAXS). Synchrotron solution-phase SAXS experiments were conducted at the 4C SAXS II beamline of the Pohang Accelerator Laboratory (Pohang, Republic of Korea). The concentration of hIAPP was adjusted to 5 mg/mL, and, for metal-included sets, 10 times higher concentration of metal chloride salts ($ZnCl_2$ or $CuCl_2$) was also added into the solution containing hIAPP. Due to the solubility of $ZnCl_2$, 100 mM Tris-HCl buffer (pH 7.5) was used for experiments, instead of 20 mM HEPES. SAXS measurements were conducted with the supernatant generated from the samples incubated for 1 h. We utilized centrifugation at 14,000 × g for 5 min to separate the soluble fraction of the oligomers. Then, the supernatant of the hIAPP solution was directly transferred to the sample cell. The sample-to-detector distances were set to 2 m and 4 m, and the magnitude of the scattering vector, q, with the range from 0.0092–0.1200 $Å^{-1}$ was used for data analysis. SAXS envelopes were generated using DAMMIN in the ATSAS 2.6.0 package (EMBL Hamburg, Germany).[2] Dimensional analyses based on the scattering profiles were performed with $p(r)$ distributions (Fig. 4) and Guinier plots (Fig. S18b).

Gel electrophoresis and Western blot. Metal-free and metal-mediated hIAPP oligomers were prepared in the same way as for the SAXS experiments. 5 μL of hIAPP incubated for 1 h was diluted five times with the sample buffer and loaded on the 10−20% Tris-tricine gel (Invitrogen, Carlsbad, CA, USA). After separation, the gel was transferred to a nitrocellulose membrane at 140 mA for 4 h and blocked with 3% (w/v) bovine serum albumin. For visualization, amylin antibody H-50 (Santa Cruz Biotechnology, Inc., Santa Cruz, CA, USA) and goat anti-rabbit IgG antibody conjugated to horseradish peroxidase (Abcam, Cambridge, UK) were used according to the manufacturers' instructions.

Metal-free hIAPP Modeling. At pH 7.5**,** hIAPP is likely to be present in the +3 charge state, with the N-terminus, Lys1, and Arg11 protonated, and His18 in its neutral form. Accordingly, a random coil structure of hIAPP in the +3 charge state was generated with HyperChem 7.5 (Hypercube Inc., Gainesville, FL, USA) and used as the initial structure for MD simulations using GROMACS 4.5.5.[3] hIAPP was modeled using the OPLS all-atom force field.[4] LINCS[5] was used to constrain all bonds. For efficient modeling of solution structures of hIAPP, replica-exchange molecular dynamics (REMD) simulation was performed with a generalized Born implicit solvent model.[6,7] For the simulation, a stochastic dynamics integrator with a time step of 2 fs was used. All nonbonded interactions were calculated, and center-of-mass translation and rotation were removed every 10 MD steps. Twelves replicas were set up at 258.50, 278.92, 300.00, 320.00, 343.51, 367.54, 392.12, 418.82, 449.13, 478.23, 511.12, and 547.39 K to obtain an average exchange probability of ~ 0.2. The systems were independently equilibrated at each temperature for 1 ns and then subjected to REMD simulations with exchanges attempted at every 2 ps intervals. Each replica was simulated for 80 ns, making the total simulation time ~ 1 μs.

Resulting structures at 300.00 K were analyzed using a clustering method in GROMACS[8] with a Cα root-mean-square-deviation (RMSD) cutoff of 0.3 nm, as has been used in a previous study on hIAPP.[9] The centroid structures of each cluster was selected as representative structures. DSSP[10] was used to analyze secondary structures of the selected structures and structures from subsequent simulations.

With the representative structures from ten largest structural clusters from REMD as initial structures, simulation of metal-free hIAPP in the gas phase was performed using 1 fs time step and the leap-frog algorithm.[11] +4 metal-free ion structures were prepared by protonating all available sites, including His18. During simulations, all nonbonded interactions were calculated and velocity rescaling thermostat[12] was used to maintain the temperature at 300 K. Center-of-mass translation and rotation were removed every 10 MD steps. Simulations were conducted for 20 ns for each of the ten systems.

Simulated annealing was performed independently to sample a wide range of hIAPP conformations in the gas phase. Overall parameters were similar to constant-temperature simulations in the gas phase, but the temperature was changed according to four temperature profiles. The four profiles were as follows 1) heat from 300 to 1500 K for 100 ps, maintain at 1500 K for 100 ps, instantly cool down to 300 K within 10 ps, and maintain at 300 K for 190 ps. 2) heat from 300 to 1200 K for 100 ps, maintain at 1200 K for 100 ps, instantly cool down to 300 K within 10 ps, and maintain at 300 K for 190 ps. 3) heat from 300 to 1500 K for 100 ps, maintain at 1500 K for 100 ps, instantly cool down to 300 K within 10 ps, and maintain at 300 K for 590 ps. 4) heat from 300 to 1500 K for 100 ps, maintain at 1500 K for 100 ps, cool down to 300 K within 100 ps, and maintain at 300 K for 100 ps. Each run was performed for 80 ns, and the final structures at the end of 300 K simulations in each cycle were collected and analyzed.

Theoretical collision cross-section (Ω_D) values in the gas phase were calculated using the exact-hard spheres scattering (EHSS) model implemented in MOBCAL[13, 14] because the projection approximation (PA) model underestimates Ω_D by 20% and the trajectory (TJ) method is computationally too expensive. Ten lowest-energy structures were selected among structures that have theoretical Ω_D within the 3% range from the experimental values.

Modeling of copper and zinc-bound hIAPP. For the copper-bound complexes, it was necessary to define a new residue type. A previous study suggested Cu(II) to be bound to hIAPP on His18 side chain, deprotonated amide nitrogen of Ser19 and Ser20, and the carbonyl oxygen or side chain oxygen of Ser20.[15] Following the well-established binding of Cu(II) to carbonyl oxygens,[16] Cu(II) was constrained to the carbonyl oxygen of Ser20 along with other three binding sites in a square planar geometry. The charges for the new residue type were adopted from a previously study on Cu(II)-bound His-Gly-Gly from octarepeat domain of the mammalian prion protein.[17] As Gly is the simplest amino acid without charged or bulky side chain, the coordination geometry and overall charge distribution of this region can reasonably be regarded to be similar to Cu(II)-bound His-Ser-Ser. Charges on Ser were slighted modified to maintain the residue neutral. For this, the charges on the two hydrogens on each Cα of Gly were averaged and distributed equally, and one of the hydrogens was replaced with the five atoms in Ser side chain with the charge on the replaced Gly hydrogen distributed equally to the new five Ser atoms. The geometry of the coordination site was adopted from a previous crystal structure on Cu(II)-bound His-Gly-Gly-Gly-Trp from the octarepeat sequence.[18] As accurate force constants for the system are not known, the bonds and angles including the copper atom were strongly fixed to a determined geometry, and dihedral restraints were additionally imposed to maintain the

geometry.

His is the only amino acid in hIAPP that typically binds Zn(II) strongly.[19] As Zn(II) is more commonly bound to the NE2 atom of His rather than to the ND1 atom,[20] we constrained a zinc atom to the NE2 atom of His. A new residue type was also defined for the Zn(II)-bound His to stably maintain the coordination site. The bond length between the zinc and NE2 atom was adopted from a previous study,[20] and bond vector was maintained by strongly fixing the angles in accordance to that of the HE2 atom on NE2. This method provided more stable coordination geometry during simulations in comparison to restraining the bond lengths between other His atoms as performed previously.[20]

Cu(II) and Zn(II) -bound structures of hIAPP were simulated in explicit solvent under periodic boundary conditions. A 2 fs time step was used for the zinc complex and a 1 fs of time step was used for stable trajectories of the copper complex. Nonbonded interactions were calculated to 1.0 nm, and particle mesh Ewald[21] was used to treat long-range electrostatics. Velocity rescaling thermostat[12] and Parrinello-Rahman barostat[22] were used to maintain the temperature and pressure at 300 K and 1 bar, respectively. The initial structures for the simulations were prepared by incorporating copper and zinc into the representative structure from the largest cluster of metal-free hIAPP from the REMD simulations described above. The peptides were solvated with TIP4P[23] water molecules. Appropriate numbers of counter ions were added to ensure overall charge neutrality of the systems. The systems were first energy minimized to remove unfavorable contacts. Then, NVT and NPT simulations with position restraints on peptides and copper/zinc were performed for 100 ps each to properly equilibrate the solvent. Copper and zinc atoms were constrained relatively weakly to the peptides during these preparation steps to prevent abrupt changes in the original peptide structure. Production runs

were performed for 50 ns.

For gas-phase simulations of Cu(II)-bound hIAPP at the +4 charge state, it was necessary to reprotonate one of the two amide nitrogens. The Ser19 amide nitrogen was reprotonated to keep a 5-membered ring, and protonation of all other available sites (the N-terminus, Lys1, and Arg11) resulted in the +4 charge state. For Zn(II)-bound hIAPP, Lys and Arg were protonated to obtain +4 charge state, and the N-terminus was kept neutral. Although little is known about the coordination properties of hIAPP in the gas phase, our ECD experiments confirm the coordination of Cu(II) and Zn(II) to His18. Furthermore, our experiments with different metal ions (Fig S3) show that electrostatic effects are most important in the gas phase, which suggest that accurate description of coordination geometry is not likely be important. Constant-temperature simulations in the gas phase was performed analogously to metal-free hIAPP, with the final structures of 50 ns simulations in solution as the initial structures, except for Cu(II) complexes for which 0.5 fs time step was used. Simulated annealing was also performed analogously to metal-free hIAPP.

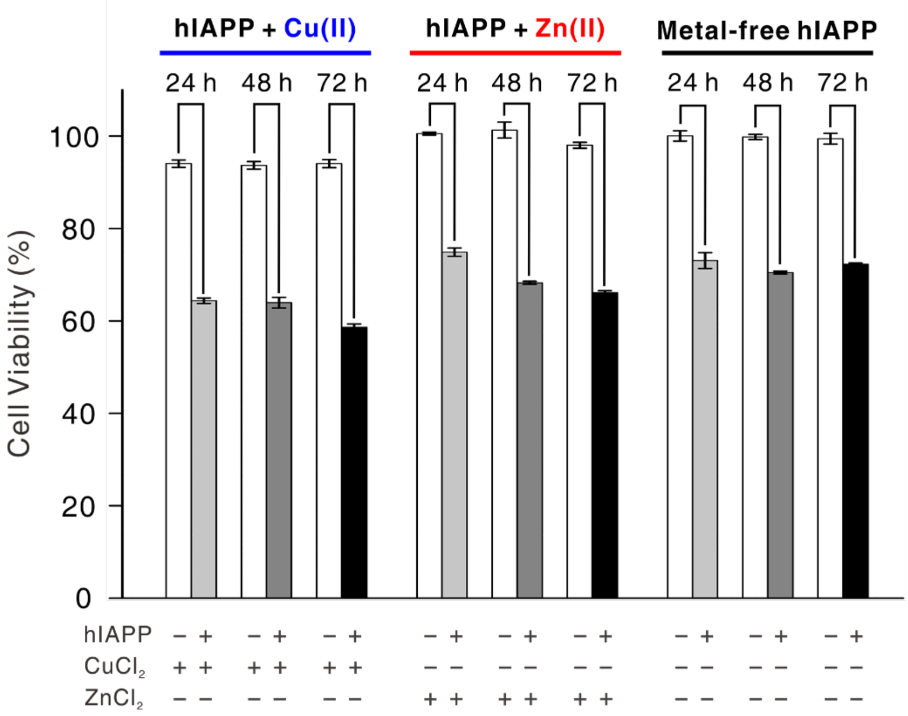

Fig. S1 Cytotoxicity of metal-free and metal-mediated hIAPP oligomers. Cell viability in this figure was normalized with water.

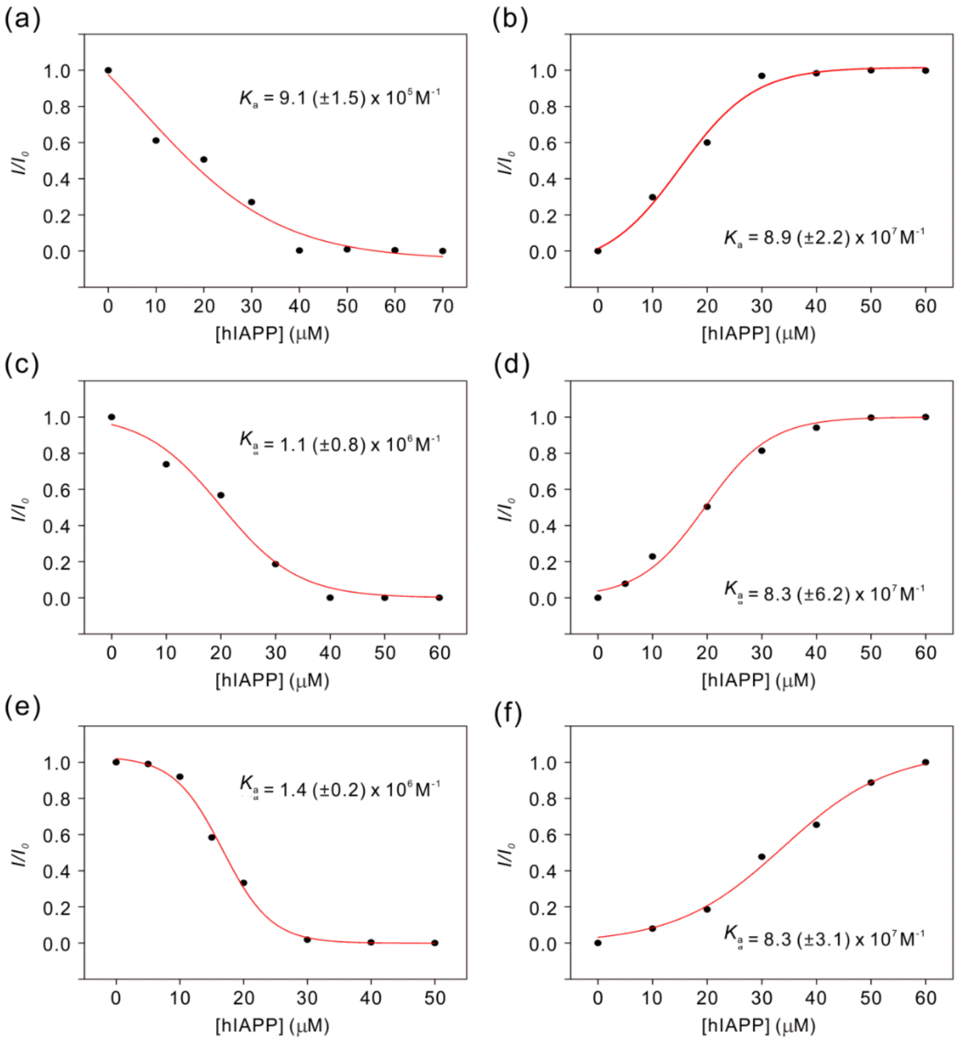

Fig. S2 Metal binding affinities of hIAPP from competition experiments with fluorescence dyes. Competition occurs between hIAPP and (a) FluoZin-3 for Zn(II), (b) FluoZin-3 for Cu(II), (c) FluoZin-1 for Zn(II), (d) Newport-Green for Cu(II) in 20 mM HEPES, pH 7.5, 20 mM NaCl, (e) FluoZin-1 for Zn(II) in 100 mM Tris-HCl, pH 7.5, and (f) Newport-Green for Cu(II) in 100 mM Tris-HCl. An equal concentration of metal-specific dyes and metal chloride salts (5 μM) was employed in the solution.

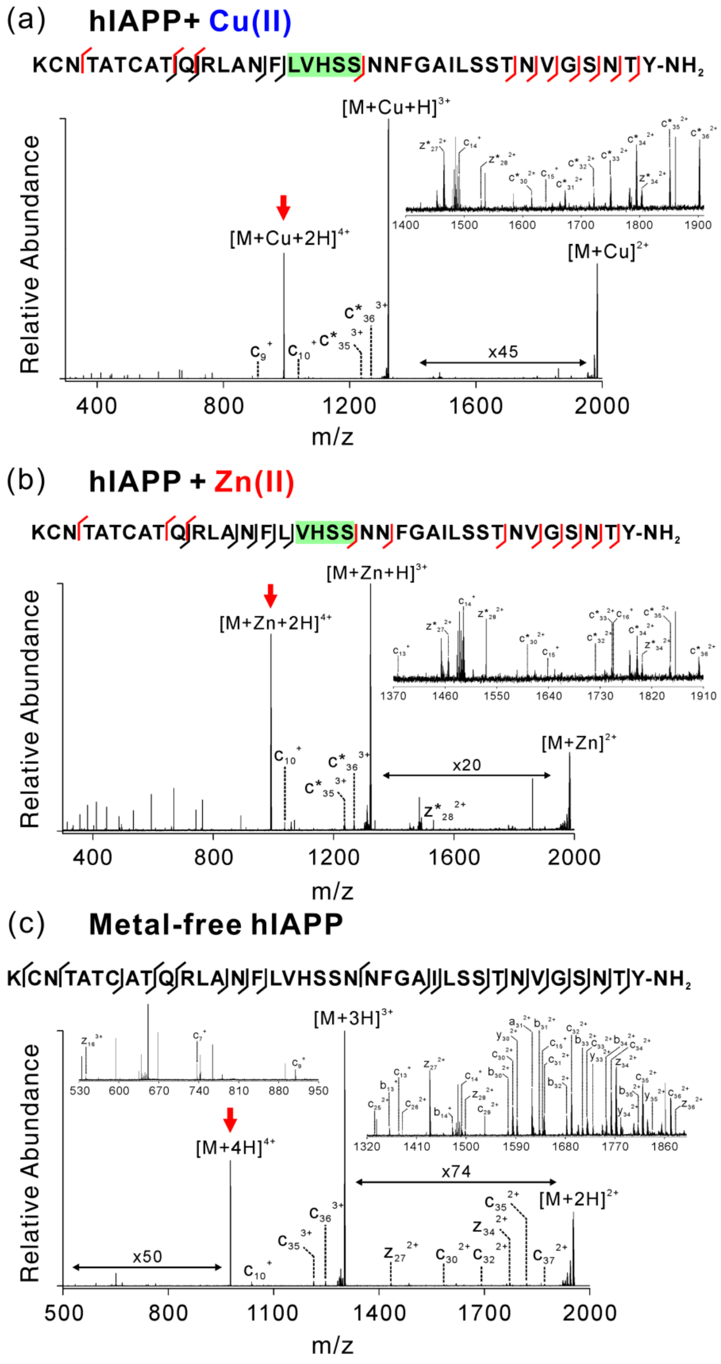

Fig. S3 Electron capture dissociation mass spectra at +4-charged (a) Cu(II)-bound hIAPP, (b) Zn(II)-bound hIAPP, and (c) metal-free hIAPP (indicated with a red arrow). Metal-bound fragment ions are denoted with asterisks(*) and colored red in the amino acid sequence. Green highlighted boxes denote the deduced potential binding sites for Cu(II) and Zn(II).

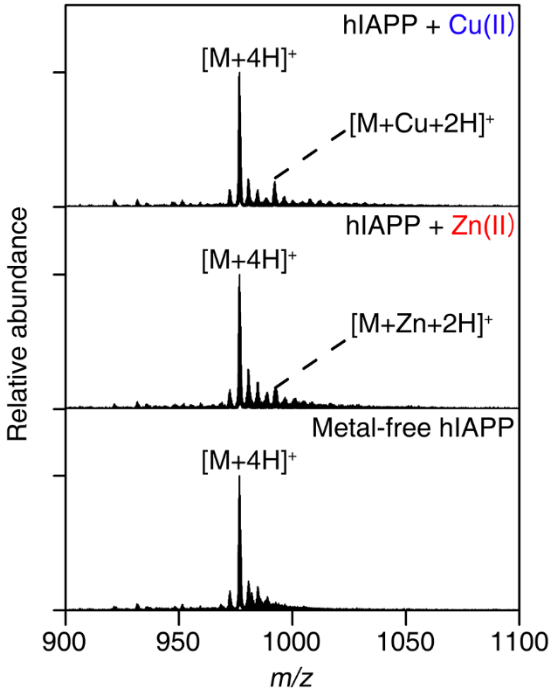

Fig. S4 Electrospray ionization mass spectra of hIAPP and metal ions in pure water (pH ~5).

(a)

Calibrants	MW (kDa)	Charge	T_D (ms)	T_D' (ms)	T_D'' (ms)	Published Ω_D (Å^2)
Melittin	2.8	3	10.91	10.87	5.59	581
		4	6.73	6.69	5.71	588
hIAPP	3.9	3	11.80	11.75	5.83	630
		4	7.39	7.35	6.00	653
			10.14	10.10	7.15	770
Insulin	5.8	3	16.87	16.81	7.10	757
		4	10.69	10.64	7.36	772
Ubiquitin	8.5	4	16.36	16.30	9.31	972

(b)

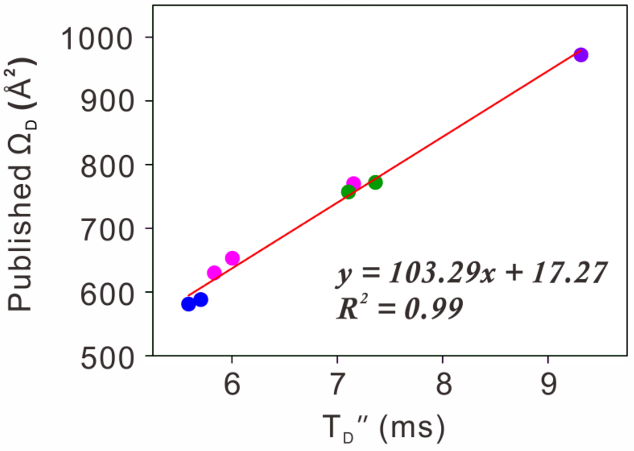

Fig. S5 Calibration for experimental collision cross-sections ($\Omega_{D,exp}$) of monomeric hIAPP. (a) Experimental drift time values of calibrants and (b) the calibration plot. Drift time values were measured with calibrant proteins (10 μM) in 20 mM ammonium acetate (pH 7.5). All procedures followed a previously described method,[1] and published Ω_D were obtained from previous reports.[9,24]

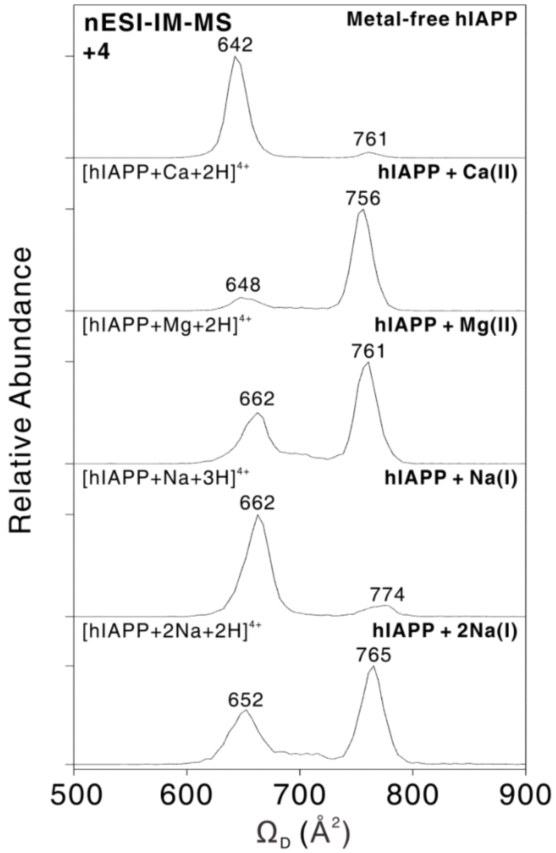

Fig. S6 ESI-IM-MS spectra of 4+ metal (Ca^{2+}, Mg^{2+}, Na^{+}, and $2Na^{+}$)-bound hIAPP ions. +4-charged hIAPP with Ca(II) and Mg(II), presents higher abundances of the extended conformer with large collision cross-section. Particularly, this phenomenon is found in the spectrum of the disodiated hIAPP but not in that of the monosodiated ion. This supports that the extended conformational distribution is partially originated from charge-charge repulsion by the introduction of the metal ion to the basic protein.

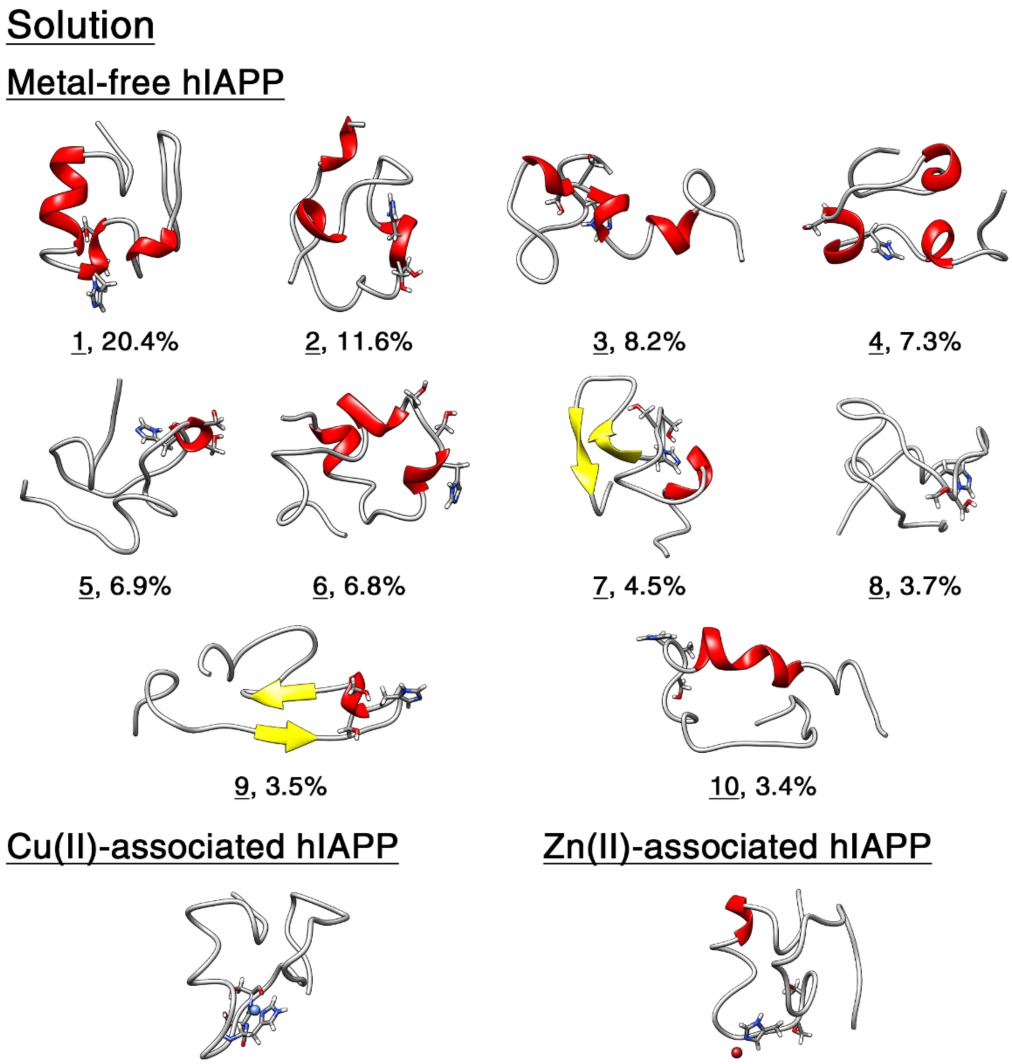

Fig. S7 Solution-phase structures of metal-free and metal-bound hIAPP monomers. The structures for the metal-free hIAPP are from REMD simulations, and the metal-bound hIAPP structures are from 50 ns NPT simulations of a representative structure from the REMD simulations of metal-free hIAPP. Atoms in His18, Ser19, and Ser20 residues are shown in the figures.

Table S1. Abundance of structural clusters in Fig. S7

Cluster #	Number of structures in cluster	Abundance
1	3266	20.4%
2	1851	11.6%
3	1306	8.2%
4	1171	7.3%
5	1108	6.9%
6	1086	6.8%
7	727	4.5%
8	585	3.7%
9	564	3.5%
10	543	3.4%
Others	517	23.7%

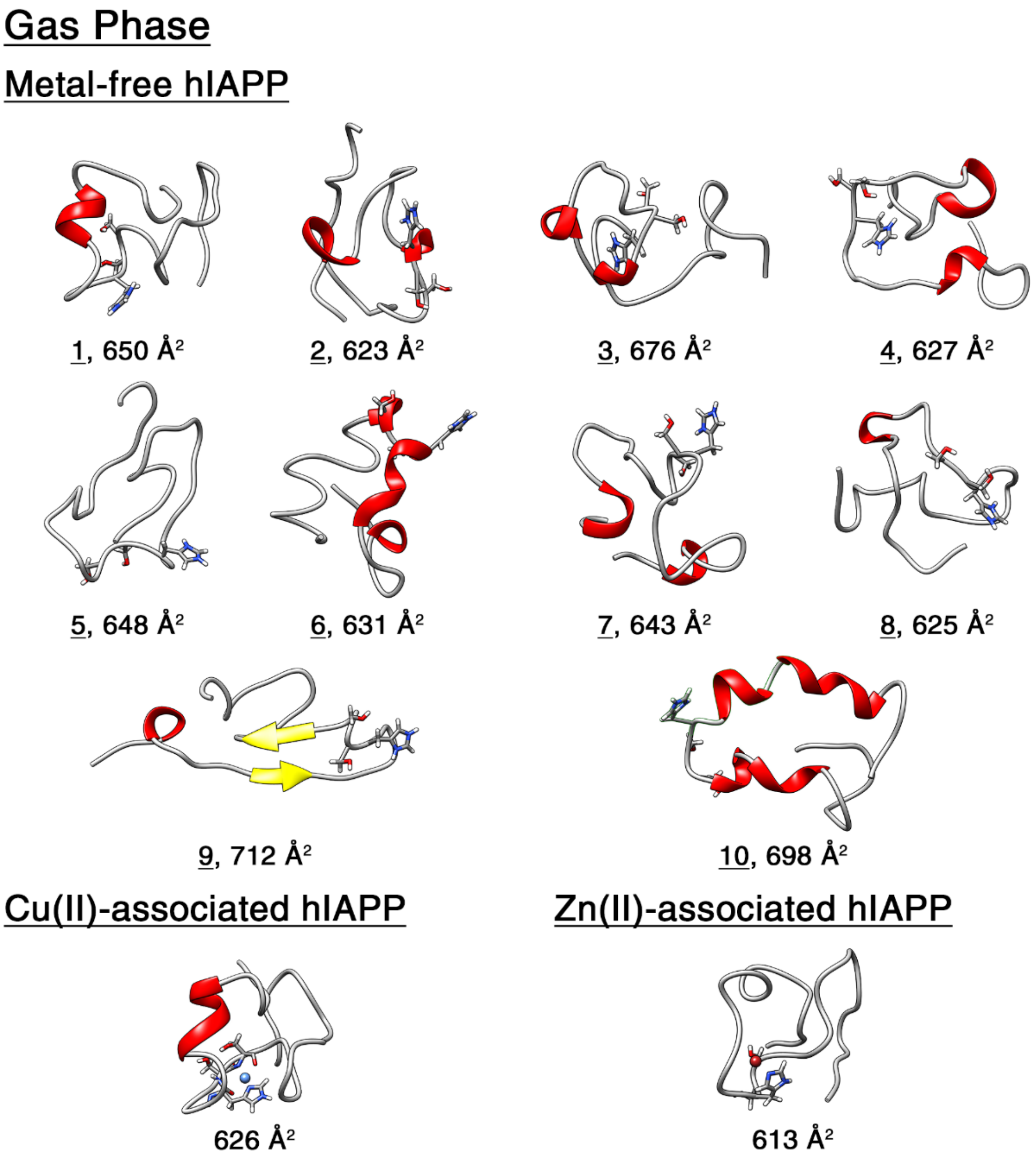

Fig. S8 Gas-phase structures of metal-free and metal-bound hIAPP monomers from 20 ns constant-temperature simulations *in vacuo*. Representative structures from REMD simulations, and final structures form 50 ns NPT simulations were used as initial structures for the metal-free and metal-bound hIAPP, respectively. Atoms in His18, Ser19, and Ser20 residues are shown in the figures.

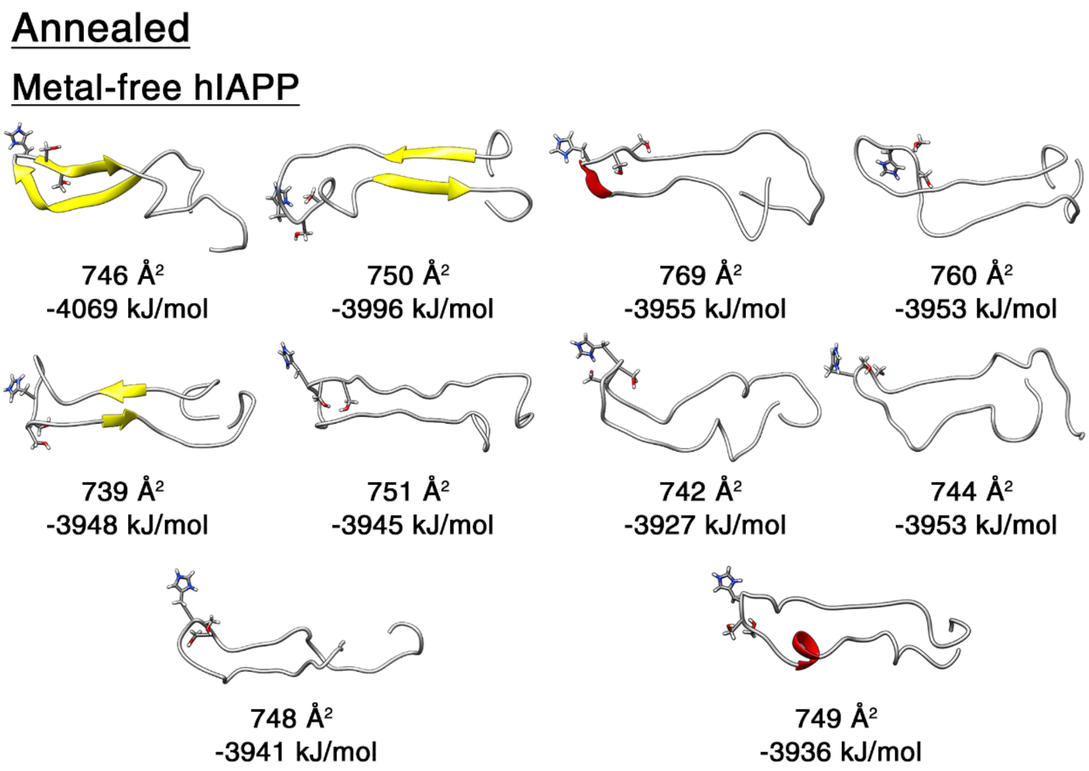

Fig. S9 Gas-phase structures of metal-free hIAPP monomers from simulated annealing *in vacuo*. Atoms in His18, Ser19, and Ser20 residues are shown in the figures.

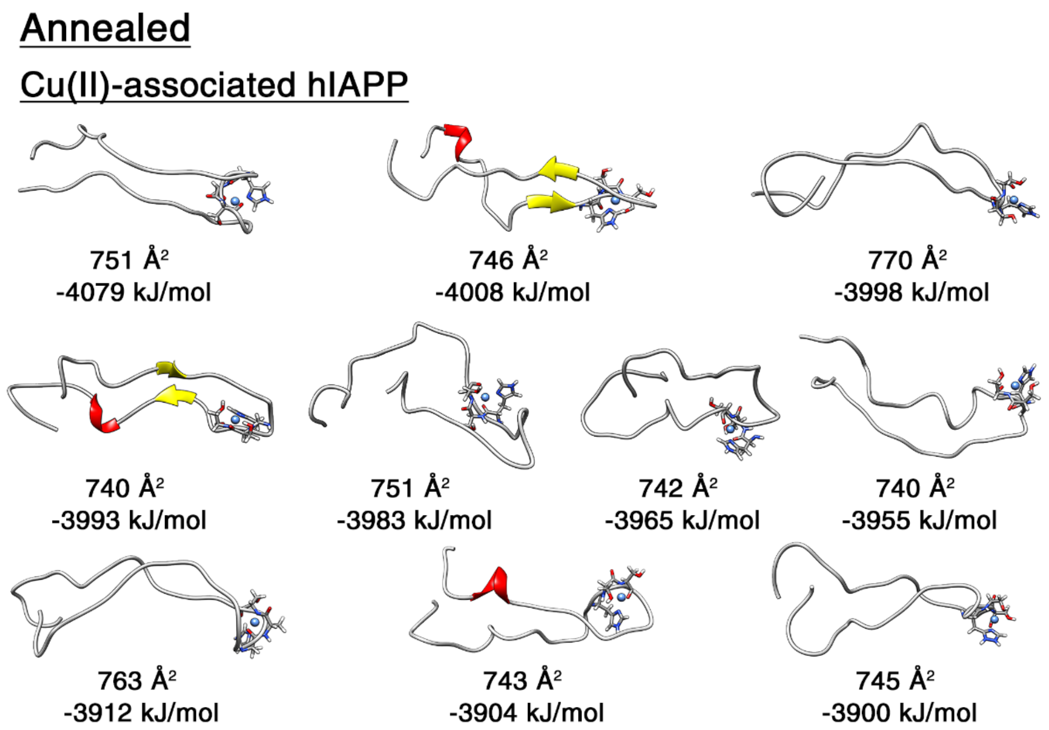

Fig. S10 Gas-phase structures of Cu(II)-bound hIAPP monomers from simulated annealing *in vacuo*. Atoms in His18, Ser19, and Ser20 residues are shown in the figures.

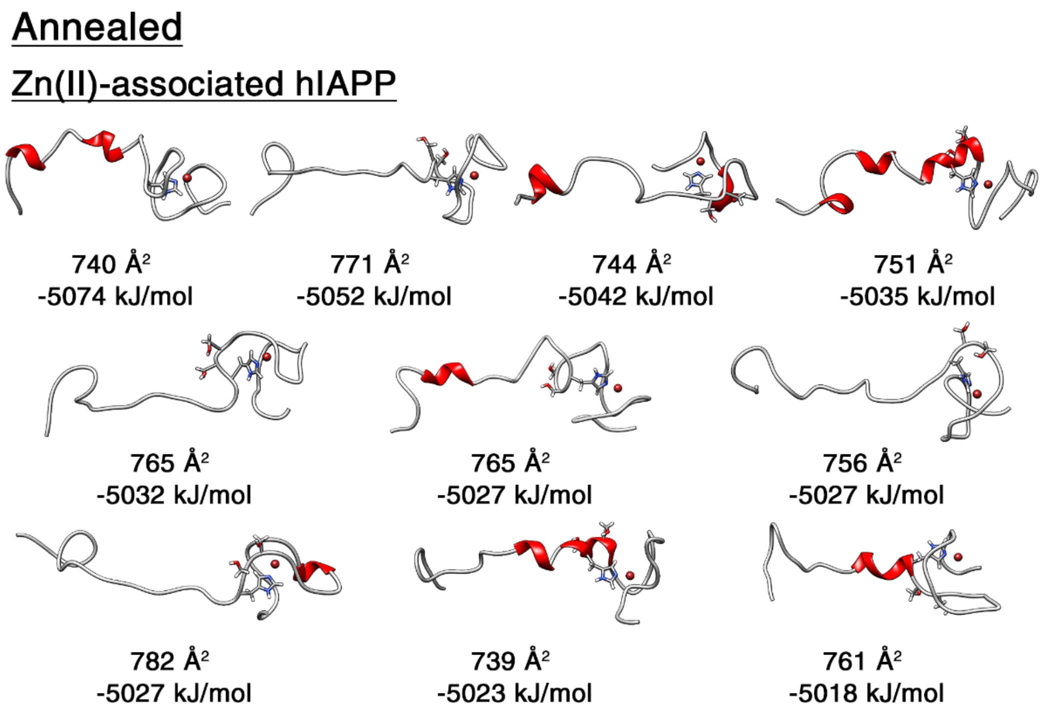

Fig. S11 Gas-phase structures of Zn(II)-bound hIAPP monomers from simulated annealing *in vacuo*. Atoms in His18, Ser19, and Ser20 residues are shown in the figures.

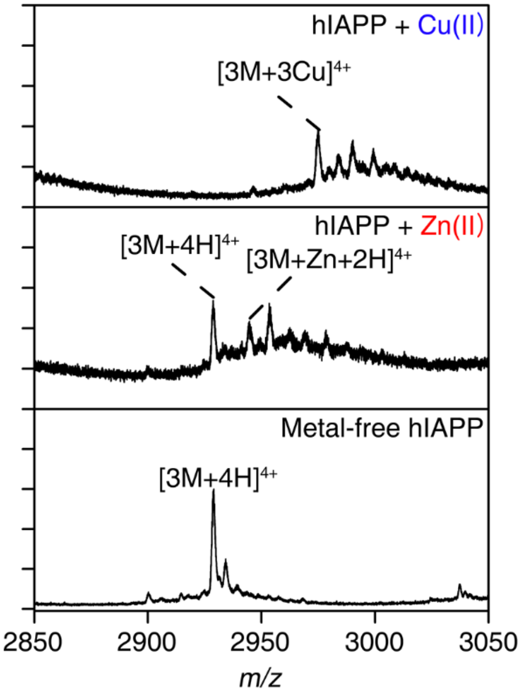

Fig. S12 Representative mass spectra for the assignment of hIAPP oligomers. We assigned metal-specific oligomer peaks with high abundance.

Table S2 Fitting parameters for the oligomer assembly models.

	Linear, $y=ax+b$			Two-thirds power, $y=ax^b$		
	a	b	R^2	a	b	R^2
hIAPP + Cu (II)	280	363	0.9924	587	0.6837	0.9999
hIAPP + Zn (II)	308	303	0.9914	589	0.6809	0.9999
Metal-free hIAPP	307	303	0.9926	588	0.6806	0.9998

(a)

Calibrants	MW (kDa)	Charge	T_D (ms)	T_D' (ms)	T_D'' (ms)	Published Ω_D (Å^2)
hIAPP	3.9	3	6.50	6.45	5.12	630
		4	4.42	4.38	5.29	653
			5.81	5.77	6.35	770
Insulin monomer	5.8	3	9.12	9.06	6.41	757
		4	5.94	5.89	6.43	772
Ubiquitin	8.5	4	8.99	8.92	8.46	972
Insulin dimer	11.6	5	8.85	8.78	10.46	1260
Cytochrome C	12.3	6	8.02	7.96	11.77	1490
β-Lactoglobulin	18.4	7	10.51	10.44	16.42	1950
Insulin hexamer	34.8	9	9.26	9.17	19.38	2440
β-Lactoglobulin dimer	36.8	11	10.09	10.01	25.09	3230
		12	10.37	10.29	27.88	3310
		13	10.09	10.02	29.66	3430
Bovine Serum Albumin	66.5	15	9.95	9.86	33.87	4490
		16	9.40	9.31	34.79	4470
		17	9.40	9.31	36.98	4490

(b)

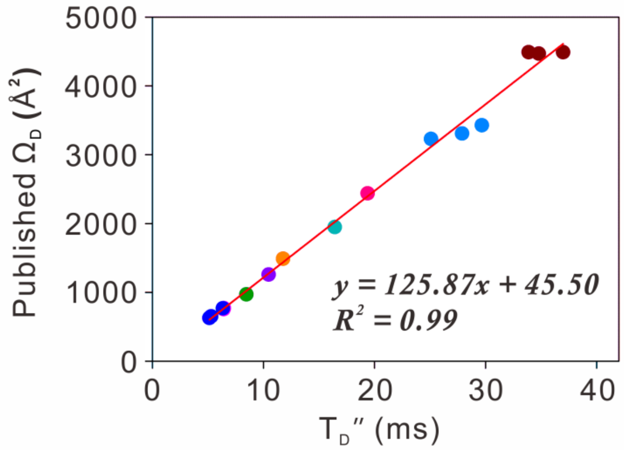

Fig. S13 Calibration for $\Omega_{D,exp}$ of oligomeric hIAPP. (a) Drift time values of calibrants (prepared as 10 μM in 20 mM ammonium acetate, pH 7.5), and (b) the calibration plot of calibrants. The calibration method is identical to that of the monomeric hIAPP, and published Ω_D were obtained from previous reports.[24,25]

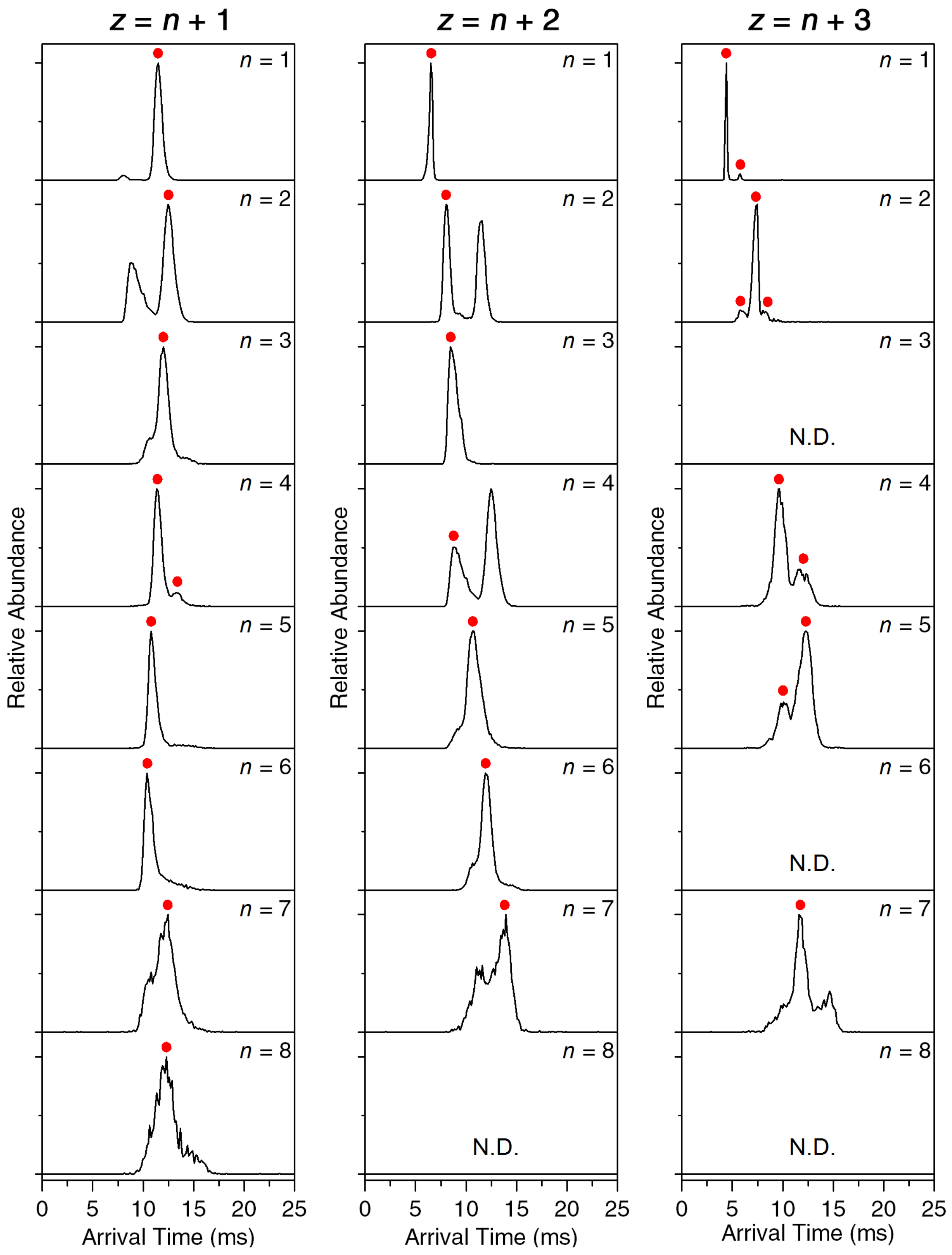

Fig. S14 Ion mobility spectra of metal-free hIAPP monomer and oligomer ions with different charge states (*z*) and oligomeric states (*n*). Relevant peaks are denoted with a red dot, and other peaks and distributions are from overlapping ions at identical *m*/*z* or noises.

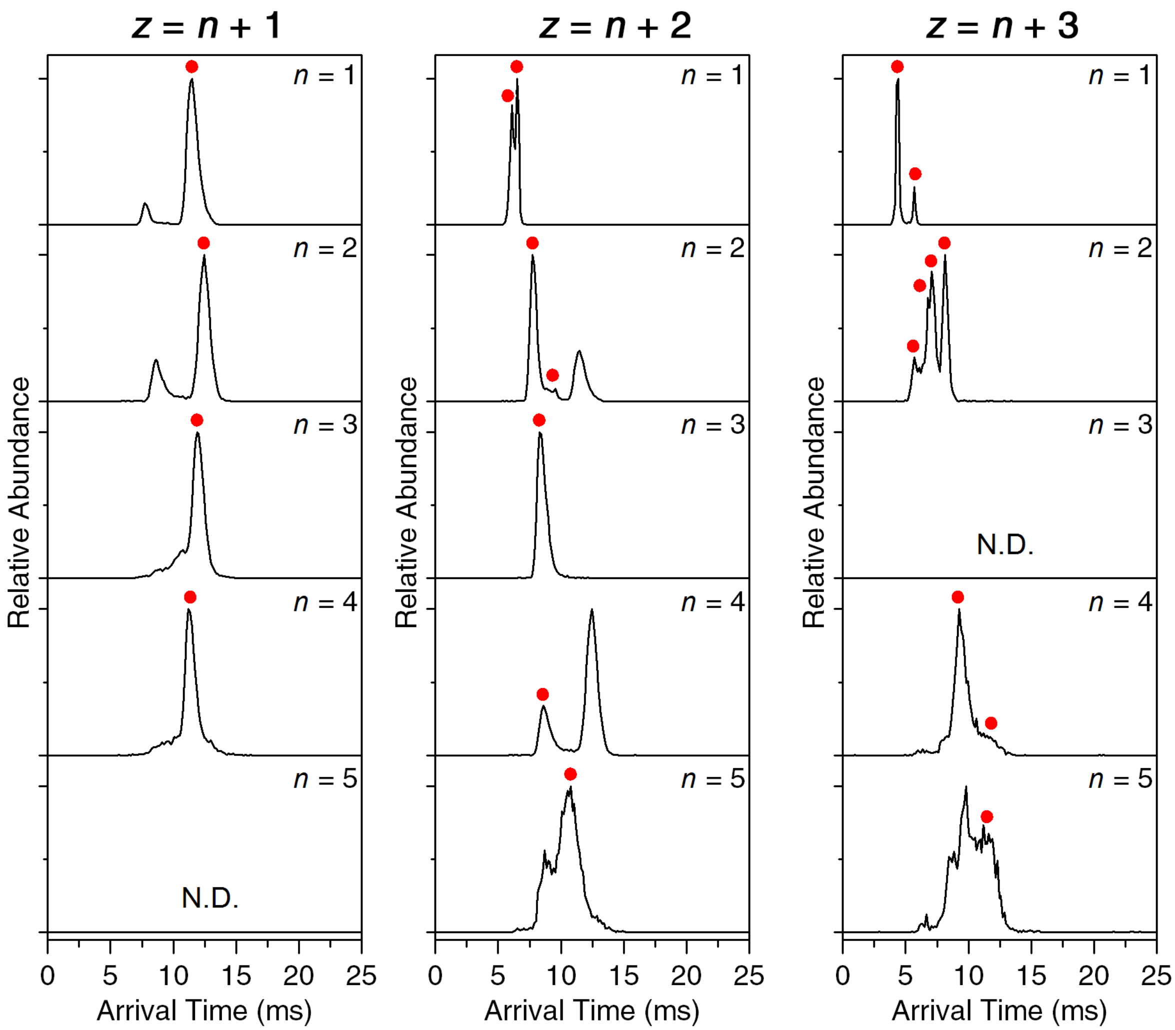

Fig. S15 Ion mobility spectra of Cu(II)-bound hIAPP monomer and oligomer ions with different charge states (z) and oligomeric states (n). Relevant peaks are denoted with a red dot, and other peaks and distributions are from overlapping ions at identical m/z or noises. The number of Cu(II) ions are identical to the oligomeric states of hIAPP for these ions.

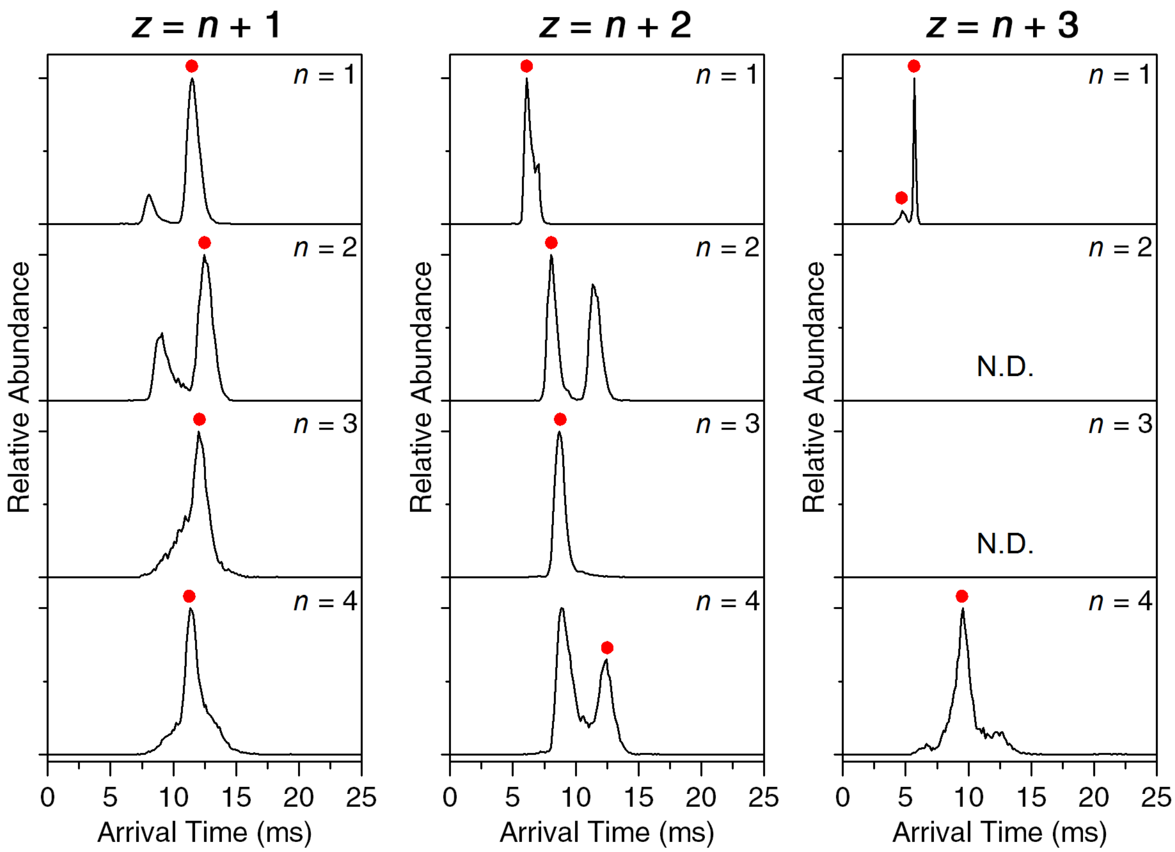

Fig. S16 Ion mobility spectra of Zn(II)-bound hIAPP monomer and oligomer ions with different charge states (z) and oligomeric states (n). Relevant peaks are denoted with a red dot, and other peaks and distributions are from overlapping ions at identical m/z or noises. Only one Zn(II) ion is bound to these ions.

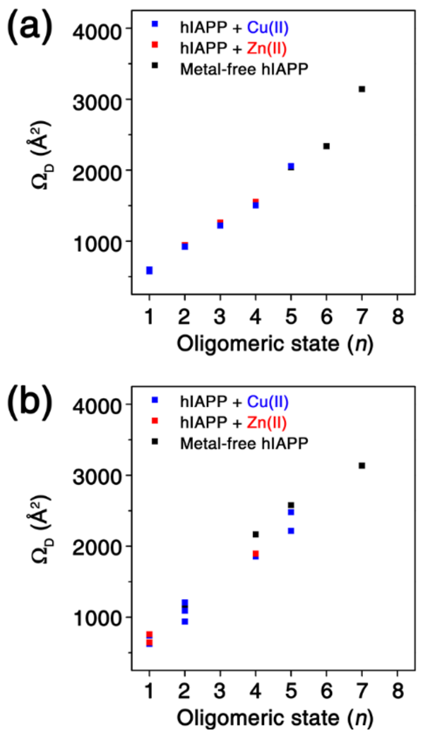

Fig. S17 Correlations between oligomeric state (n) and Ω_D of (a) n^{n+2} and (b) n^{n+3} for experimentally detected oligomers. We plotted highly abundant peaks in the arrival time distribution of hIAPP oligomers. Calibration curve of the oligomers is available in Fig. S12

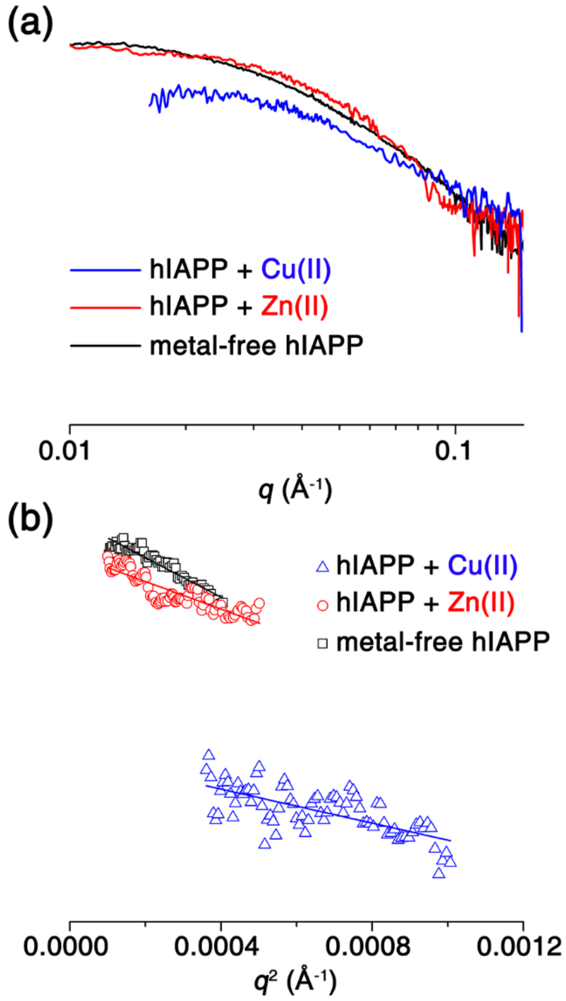

Fig. S18 (a) Scattering profiles of the supernatant from incubated hIAPP solutions. (b) Guinier fits for SAXS scattering profiles of metal-free and metal-mediated hIAPP oligomers.

References

1. B. T. Ruotolo, J. L. P. Benesch, A. M. Sandercock, S.-J. Hyung and C. V. Robinson, *Nat. Protoc.*, 2008, **3**, 1139-1152.
2. D. I. Svergun, *Biophys. J.*, 1999, **76**, 2879-2886.
3. B. Hess, C. Kutzner, D. van der Spoel and E. Lindahl, *J. Chem. Theory Comput.*, 2008, **4**, 435-447.
4. G. A. Kaminski, R. A. Friesner, J. Tirado-Rives and W. L. Jorgensen, *J. Phys. Chem. B*, 2001, **105**, 6474-6487.
5. B. Hess, H. Bekker, H. J. C. Berendsen and J. G. E. M. Fraaije, *J. Comput. Chem.*, 1997, **18**, 1463-1472.
6. A. Onufriev, D. Bashford and D. A. Case, *Proteins: Struct., Funct., Bioinf.*, 2004, **55**, 383-394.
7. M. Schaefer, C. Bartels and M. Karplus, *J. Mol. Biol.*, 1998, **284**, 835-848.
8. X. Daura, K. Gademann, B. Jaun, D. Seebach, W. F. van Gunsteren and A. E. Mark, *Angew. Chem. Int. Ed.*, 1999, **38**, 236-240.
9. N. F. Dupuis, C. Wu, J.-E. Shea and M. T. Bowers, *J. Am. Chem. Soc.*, 2009, **131**, 18283-18292.
10. W. Kabsch and C. Sander, *Biopolymers*, 1983, **22**, 2577-2637.
11. R. W. Hockney, S. P. Goel and J. W. Eastwood, *J. Comput. Phys.*, 1974, **14**, 148-158.
12. G. Bussi, D. Donadio and M. Parrinello, *J. Chem. Phys.*, 2007, **126**, 014101.
13. A. A. Shvartsburg and M. F. Jarrold, *Chem. Phys. Lett.*, 1996, **261**, 86-91.
14. M. F. Mesleh, J. M. Hunter, A. A. Shvartsburg, G. C. Schatz and M. F. Jarrold, *J. Phys. Chem.*, 1996, **100**, 16082-16086.
15. L. Rivillas-Acevedo, C. Sánchez-López, C. Amero and L. Quintanar, *Inorg. Chem.*, 2015, **54**, 3788-3796.
16. H. Sigel and R. B. Martin, *Chem. Rev.*, 1982, **82**, 385-426.
17. M. J. Pushie and H. J. Vogel, *Biophys. J.*, 2007, **93**, 3762-3774.
18. C. S. Burns, E. Aronoff-Spencer, C. M. Dunham, P. Lario, N. I. Avdievich, W. E. Antholine, M. M. Olmstead, A. Vrielink, G. J. Gerfen, J. Peisach, W. G. Scott and G. L. Millhauser, *Biochemistry*, 2002, **41**, 3991-4001.
19. D. S. Auld, *Biometals*, **14**, 271-313.
20. P. Nedumpully-Govindan, Y. Yang, R. Andorfer, W. Cao and F. Ding, *Biochemistry*, 2015, **54**, 7335-7344.
21. T. Darden, D. York and L. Pedersen, *J. Chem. Phys.*, 1993, **98**, 10089-10092.
22. M. Parrinello and A. Rahman, *J. Appl. Phys.*, 1981, **52**, 7182-7190.
23. W. L. Jorgensen, J. Chandrasekhar, J. D. Madura, R. W. Impey and M. L. Klein, *J. Chem. Phys.*, 1983, **79**, 926-935.
24. R. Salbo, M. F. Bush, H. Naver, I. Campuzano, C. V. Robinson, I. Pettersson, T. J. D. Jorgensen and K. F. Haselmann, *Rapid Comm. Mass Spectrom.*, 2012, **26**, 1181-1193.
25. M. F. Bush, Z. Hall, K. Giles, J. Hoyes, C. V. Robinson and B. T. Ruotolo, *Anal. Chem.*, 2010, **82**, 9557-9565.
